# Investigation of adrenal and thyroid gland dysfunction in dogs with ultrasonographic diagnosis of gallbladder mucocele formation

**DOI:** 10.1371/journal.pone.0212638

**Published:** 2019-02-27

**Authors:** Kathleen M. Aicher, John M. Cullen, Gabriela S. Seiler, Katharine F. Lunn, Kyle G. Mathews, Jody L. Gookin

**Affiliations:** 1 Department of Clinical Sciences, College of Veterinary Medicine and Comparative Medicine Institute, North Carolina State University, Raleigh, North Carolina, United States of America; 2 Department of Population Health and Pathobiology, College of Veterinary Medicine and Comparative Medicine Institute, North Carolina State University, Raleigh, North Carolina, United States of America; 3 Department of Molecular Biomedical Sciences, College of Veterinary Medicine and Comparative Medicine Institute, North Carolina State University, Raleigh, North Carolina, United States of America; University of Illinois, UNITED STATES

## Abstract

Gallbladder mucocele formation is an emerging disease in dogs characterized by increased secretion of condensed granules of gel-forming mucin by the gallbladder epithelium and formation of an abnormally thick mucus that can culminate in obstruction of the bile duct or rupture of the gallbladder. The disease is associated with a high morbidity and mortality and its pathogenesis is unknown. Affected dogs have a significantly increased likelihood of concurrent diagnosis of hyperadrenocorticism, hypothyroidism, and hyperlipidemia. Whether these endocrinopathies represent coincidental primary disease processes that exacerbate gallbladder mucocele formation in predisposed dogs or reflect a concurrent disruption of endocrine and lipid metabolism is unclear. In this study, we investigated a hypothesis that dogs with gallbladder mucocele formation would have a high prevalence of occult and atypical abnormalities in adrenal cortical and thyroid gland function that would suggest the presence of endocrine disruption and provide deeper insight into disease pathogenesis. We performed a case-control study of dogs with and without ultrasonographic diagnosis of gallbladder mucocele formation and profiled adrenal cortical function using a quantitative mass spectrometry-based assay of serum adrenal-origin steroids before and after administration of synthetic cosyntropin. We simultaneously profiled serum thyroid hormone concentrations and evaluated iodine sufficiency by measurement of urine iodine:creatinine ratios (UICR). The studies were complemented by histological examination of archival thyroid tissue and measurements of thyroid gland organic iodine from dogs with gallbladder mucocele formation and control dogs. Dogs with gallbladder mucocele formation demonstrated an exaggerated cortisol response to adrenal stimulation with cosyntropin. A prevalence of 10% of dogs with gallbladder mucocele formation met laboratory-based criteria for suspect or definitive diagnosis of hyperadrenocorticism. A significantly greater number of dogs with gallbladder mucocele formation had basal serum dehydroepiandrosterone (DHEAS) increases compared to control dogs. A high percentage of dogs with gallbladder mucocele formation (26%) met laboratory-based criteria for diagnosis of hypothyroidism, but lacked detection of anti-thyroglobulin antibodies. Dogs with gallbladder mucocele formation had significantly higher UICRs than control dogs. Examination of thyroid tissue from an unrelated group of dogs with gallbladder mucocele formation did not demonstrate histological evidence of thyroiditis or significant differences in content of organic iodine. These findings suggest that dogs with gallbladder mucocele formation have a greater capacity for cortisol synthesis and pinpoint DHEAS elevations as a potential clue to the underlying pathogenesis of the disease. A high prevalence of thyroid dysfunction with absent evidence for autoimmune thyroiditis suggest a disrupted thyroid hormone metabolism in dogs with gallbladder mucocele formation although an influence of non-thyroidal illness cannot be excluded. High UICR in dogs with gallbladder mucocele formation is of undetermined significance, but of interest for further study.

## Introduction

Gallbladder mucocele formation is an emerging disease in dogs. The disease is characterized by increased secretion of condensed granules of gel-forming mucin[1] by the gallbladder epithelium and formation of an abnormally thick mucus that can result in impaired gallbladder motility, extrahepatic biliary tract obstruction, and gallbladder rupture with bile peritonitis [2–13]. For clinically affected dogs, surgery to remove the gallbladder can be life-saving. However, retrospective studies report that a median of 27% (range from 7 to 45%)[3–10] of dogs will die or be euthanized within 2 weeks of hospitalization due to post-operative complications. Diagnosis of gallbladder mucocele formation was rare as recently as 15 years ago[14, 15]. Gallbladder mucocele formation is now regarded as one of the most common and poorly understood biliary diseases of dogs[3–13].

The cause of gallbladder mucocele formation in dogs is unknown and likely multifactorial. There are no reported gallbladder diseases in humans that match the gross or histological description of gallbladder mucocele formation in dogs. The disease has a strong predilection for purebred dog such as the Shetland Sheepdog, Border Terrier, Cocker Spaniel, Miniature Schnauzer, Pomeranian, Chihuahua, and others[5–7, 16–18] but is diagnosed in older-aged dogs which suggests an influence of both genetic predisposition and time. An interesting observation in dogs with gallbladder mucocele formation is an increased likelihood for concurrent diagnosis of hyperadrenocorticism, hypothyroidism, and hyperlipidemia [5, 13, 16, 17, 19]. These diagnoses are not uncommon in dogs, however their association with gallbladder mucocele formation is recognized as new [5, 13, 16, 17]. It is possible that hyperadrenocorticism, hypothyroidism, and hyperlipidemia are coincidental primary disease processes that exacerbate gallbladder mucocele formation in predisposed dogs. On the other hand, the underlying cause of gallbladder mucocele formation may be responsible for concurrent disruption of endocrine and lipid metabolism in these dogs. Efforts to determine if dogs with gallbladder mucocele formation have some form of endocrine disruption has important implications for explaining the as-yet unknown pathogenesis of the disease.

Several observations deepen an interest in adrenal and thyroid gland function in dogs with mucocele formation. The first is that pathological findings similar to gallbladder mucocele formation are described in studies examining the toxicological effect of estrogen and progestogen treatment in dogs [20–24] and several Shetland sheepdogs with gallbladder disease have been described as having “atypical” hyperadrenocorticism characterized by high serum concentrations of progesterone [5]. The second observation is that gallbladder mucocele formation in dogs has a strong resemblance to descriptions of the gallbladder in piglets and ferrets with cystic fibrosis caused by mutation of the cystic fibrosis transmembrane conductance regulator protein gene (CFTR)[25, 26]. In patients with cystic fibrosis, subclinical hypothyroidism was historically associated with iodine excess (i.e. use of iodine-containing expectorants)[27] but can also be associated with iodine deficiency[28]. While the mechanism is still unclear, CFTR is expressed by thyroid follicular cells[29] where it is suspected to provide an ion exchange mechanism necessary for function of the iodide transporter pendrin[30]. Abnormal iodide uptake results in impaired thyroid hormone synthesis[31].

There is strong circumstantial evidence that hyperadrenocorticism and hypothyroidism, regardless of their pathogenesis, may promote the progression of gallbladder mucocele formation by contributing to impaired gallbladder motility[2, 5], altering the composition of gallbladder bile acids[32], or increasing the quantity of gallbladder sludge[33]. In 2 dogs, treatment of concurrent hypothyroidism was described to result in medical resolution of gallbladder mucocele formation[34]. Given the possibility of a therapeutic benefit to treating underlying hypothyroidism or hyperadrenocorticism in dogs with gallbladder mucocele formation, clinicians may be more likely to test for these endocrinopathies even in dogs that lack clinical signs of endocrine disease. Therefore, it is also of interest to describe what these test results are likely to yield if performed in this population and to determine any correlations between serum biochemistry findings and results of endocrine test results when performed simultaneously in dogs with gallbladder mucocele formation.

The objective of this prospective case-controlled study was to determine the prevalence of occult or atypical abnormalities in adrenal cortical and thyroid gland function in dogs that lack clinical signs or physical examination findings suggestive of endocrinopathy at the time of diagnosis of gallbladder mucocele formation. We sought to ascertain if identified abnormalities are consistent with laboratory-based criteria for diagnosis of hyperadrenocorticism or hypothyroidism, expected changes in thyroid hormone concentrations in dogs with concurrent illness, or suggestive of an effect of endocrine disruption or iodine deficiency. Accordingly, our approach was to utilize a quantitative mass spectrometry-based assay to measure a panel of adrenal-origin steroids before and after ACTH stimulation testing using synthetic cosyntropin. We simultaneously profiled serum thyroid hormone concentrations and urine iodine:creatinine ratios in control dogs and dogs with gallbladder mucocele formation. These studies were complemented by histological examination of archival thyroid tissue from dogs with gallbladder mucocele formation and measurements of thyroid gland iodide. Finally, we analyzed the adrenal and thyroid hormone test results of these dogs for significant correlations with results of simultaneous serum biochemistry profile testing.

## Methods

### Case-control study

#### Patient recruitment and inclusion criteria

Client-owned dogs with presumptive diagnosis of gallbladder mucocele formation at the North Carolina State University Veterinary Hospital (NCSU-VH) were prospectively identified for possible inclusion into the study over the time period from February 2014 to January 2017. In each case, diagnosis of gallbladder mucocele formation was confirmed by a single board-certified veterinary radiologist (G.S.) based on previously published ultrasonographic criteria[9]. These criteria included an enlarged gallbladder containing non-gravity dependent, immobile bile having hypoechoic extensions of mucus into the lumen, resulting in a stellate or finely striated bile pattern. In the event that the dog was euthanized or underwent surgery for removal of the gallbladder, the gross pathology and histopathology reports were reviewed to confirm the ultrasonographic diagnosis of gallbladder mucocele formation.

An apparently healthy, age, breed, and sex-matched cohort of client-owned dogs were concurrently recruited by the Clinical Studies Core facility at North Carolina State University for inclusion as controls. For each control dog, ultrasonography was used to confirm absence of gallbladder mucocele formation based on a normal appearing gallbladder with normal wall structure and thickness. Sludge, if present, was gravity-dependent, occupied less than 50% of the gallbladder lumen, and was not attached to the wall.

Dogs were excluded if they had previously been diagnosed with, treated for, or suspected, based on clinical signs or physical examination findings, of having hypothyroidism or hyperadrenocorticism. Dogs were excluded if they had a recent (within 2 months) history of treatment with ursodeoxycholic acid or drugs recognized or suspected to interfere with thyroid or adrenal function testing (e.g. topical or systemic steroids, non-steroidal anti-inflammatory drugs, anti-convulsants, furosemide, sulfa-containing drugs, fatty acid supplements) or were reproductively intact. Because prior studies have not demonstrated any significant association between gallbladder mucocele formation and intact reproductive status, reproductively intact dogs were excluded from the study to eliminate a confounding influence of gonadal steroidogenesis[35, 36]. Owners of each dog signed an informed consent for participation in the study. All study protocols were approved by the Institutional Animal Care and Use Committee of North Carolina State University (ID#14-049-O).

#### Sample collection and corticotropin stimulation testing

Upon enrollment into the study, dogs diagnosed with gallbladder mucocele formation and control dogs underwent a complete physical examination by the attending clinician. Blood was collected by means of venipuncture and urine was collected by ultrasound-guided cystocentesis. Samples were obtained after a minimum fasting period of 12 hours. Time of day of sampling was dictated by patient accessibility and therefore not standardized. Anticoagulated (EDTA) whole blood, plasma, serum, and urine were processed by the NCSU-VH Clinical Pathology Laboratory for a complete blood cell count and serum biochemical analysis. Aliquots of plasma, serum, and urine were also stored at -80°C within 30-min of collection. Each dog received an intravenous injection of synthetic cosyntropin (Cortrosyn, Amphastar Pharmaceuticals, Inc, Rancho Cucamonga, CA) according to the following dose regimen: ≤ 5 kg = 25 μg, 5.1–10 kg = 50 μg, 10.1–15 kg = 75 μg, 15.1–20.0 kg = 100 μg, and 20–50 kg = 250 μg. One hour following administration of cosyntropin, a second blood sample was drawn and plasma and serum were stored at -80°C within 30-min of collection. Plasma endogenous adrenocorticotropic hormone (ACTH) concentration was measured in samples that were obtained pre-cosyntropin administration from each dog. Plasma for ACTH measurement was separated from EDTA-anticoagulated whole blood by centrifugation at 2,000 x g for 10-minutes under refrigeration (5°C). Each plasma sample was subsequently frozen and stored at -80°C until the time of batch testing using a previously validated assay (IMMULITE 1000 Canine ACTH; Siemens Healthcare Diagnostics, Llanberis, Gwynedd, UK[37]).

#### Targeted mass spectrometric analysis of serum steroids

Frozen pre- and post-cosyntropin serum samples were shipped on 20 kg of dry ice to a commercial laboratory (BIOCRATES Life Sciences AG, Innsbruck, Austria) and confirmed to arrive frozen. Analysis of 17 different steroid hormones was performed using a standardized Ultra High Performance Liquid Chromatography tandem mass spectrometry-based quantitative multiplex assay (Absolute*IDQ* Stero17 Kit, BIOCRATES Life Sciences AG) that has undergone an extensive validation process for use in humans [38]. For quantification of each steroid compound, 7-point calibration curves and 13 stable isotope-labeled internal standards were used. Compounds analyzed included 11-deoxycorticosterone (limit of detection (LOD), 0.01 nM), 11-deoxycortisol (LOD, 0.01 nM), corticosterone (LOD, 0.01 nM), cortisol (LOD, 0.3907 nM), cortisone (LOD, 0.038 nM), progesterone (LOD, 0.016 nM), 17α-hydroxyprogesterone (LOD, 0.017 nM), androstenedione (LOD, 0.011 nM), testosterone (LOD, 0.01 nM), aldosterone (LOD, 0.0828 nM), dehydroepiandrosterone sulfate (DHEAS) (LOD, 39 nM), dihydrotestosterone (LOD, 0.01 nM), androsterone (LOD, 0.022 nM), estrone (LOD, 0.019 nM), estradiol (LOD, 0.01 nM), etiocholanolone (LOD, 0.0517 nM), and dehydroepiandrosterone (LOD, 0.056 nM).

#### Thyroid hormone assays

Serum samples were obtained from all dogs prior to cosyntropin administration, stored at -80°C and collectively submitted on dry ice to a commercial laboratory (Michigan State University Veterinary Diagnostic Laboratory, Endocrinology Section, Lansing, MI) for measurement of total thyroxine (T4), total triiodothyronine (T3), free thyroxine by equilibrium dialysis (FT_4_), free triiodothyronine (FT_3_), thyrotropin (TSH), and antibodies against thyroxine (T4AA), triiodothyronine (T3AA), and thyroglobulin (TgAA) using methods previously described and validated for use in dogs[39–43]. Briefly, serum T4 (T4 MAb Solid Phase Component System; MP Biomedicals, Diagnostics Division, Orangeburg, NY), FT3 (Free T_3_ Solid Phase Component System; MP Biomedicals, Diagnostics Division, Orangeburg, NY), T4AA, and T3AA concentrations were measured by radioimmunoassay. Serum T3 concentrations were measured using an in-house charcoal-separation radioimmunoassay. Serum FT_4_ concentrations were measured by equilibrium dialysis in combination with radioimmunoassay (Free T_4_ by Equilibrium dialysis; Antech Diagnostics, Irvine, CA). Serum TSH concentrations were measured by use of a solid-phase chemiluminescent immunometric assay (IMMULITE 2000 Canine TSH; Siemens Healthcare Diagnostics, Llanberis, Gwynedd, UK). Serum concentrations of canine TgAA were measured with an enzyme-linked immunosorbent assay (Canine thyroglobulin autoantibody ELISA; Oxford Biomedical Research, Oxford, MI) properly blanked for nonspecific binding. Results of TGAA were qualitatively determined as positive (>35%), inconclusive (20–35%), or negative (<20%).

#### Quantification of inorganic iodine in urine

Urine samples were collected from each dog prior to cosyntropin administration, stored at -80°C, and then collectively submitted to a commercial laboratory (Michigan State University Veterinary Diagnostic Laboratory, Nutrition Section, Lansing, MI) for measurement of inorganic iodine by means of inductively-coupled plasma mass spectrometry (ICP-MS). Urine creatinine was measured using a commercially available chemistry analyzer (Roche Cobas c501 Chemistry system; Roche Diagnostics USA) and used to calculate a urine inorganic iodine:creatinine ratio (UICR; μg/g) for each dog.

#### Scoring of clinical illness severity

To evaluate thyroid hormone test results for an influence of non-thyroidal illness (NTI), all dogs were stratified by disease severity into four groups based on a previously described scoring system[44] as follows: absent (0) for patients that demonstrated no clinical signs of illness, mild (1) for patients with signs of clinical disease but suitable for outpatient care, moderate (2) for patients sick enough to require hospitalization and aggressive treatment, and severe (3) for patients with severe illness requiring intensive care and advanced treatment (including all dogs requiring emergency cholecystectomy).

### Examination of thyroid glands from unrelated groups of dogs undergoing post-mortem examination

#### Quantification of organic iodine in thyroid tissue obtained opportunistically from euthanized dogs with and without gallbladder mucocele formation

Fresh thyroid tissue was collected by a study investigator (J.L.G.) from dogs diagnosed with a gallbladder mucocele that underwent euthanasia during the time course of this study. Thyroid glands were likewise obtained from an unmatched group of apparently healthy shelter and research dogs that underwent euthanasia during the time course of the study. All control dogs lacked gallbladder mucocele formation confirmed by gross inspection of the gallbladder contents. Both thyroid lobes were removed and each divided transversely into two halves with a scalpel blade. One half of each lobe was placed in a 1.7 ml microcentrifuge tube and frozen at -80°C until analysis. Quantification of organic iodine was performed in a commercial laboratory (Michigan State University Veterinary Diagnostic Laboratory, Nutrition Section, Lansing, MI) by means of ICP-MS and reported as μg/g dry weight.

#### Retrospective light microscopic and histomorphometric examination of archival thyroid tissue from dogs with and without gallbladder mucocele formation

The electronic medical records database of the NCSU-VH was searched over the time interval from 2002 to 2015 to identify all dogs having a histological diagnosis of gallbladder mucocele formation using previously described criteria[17] and from whom thyroid tissue was archived at the time of post-mortem examination. Two groups of control dogs were also identified. The first control group consisted of adult dogs of breeds with known predisposition to gallbladder mucocele formation but having histologically normal appearing gallbladders and from whom thyroid tissue was also archived at the time of post-mortem examination. The second control group consisted of apparently healthy research and shelter dogs from which convenience samples of gallbladder and thyroid glands were prospectively obtained for this study at the time of euthanasia. Dogs were excluded if they had known history of diagnosis or treatment for hypothyroidism.

Formalin-fixed and paraffin-embedded thyroid tissue was sectioned at a thickness of 5 μm, mounted onto glass microscopic slides and stained with Mayer-Harris hematoxylin and alcoholic eosin Y. Each slide was examined by a single board-certified veterinary pathologist (J.C.) who was blinded to the identities of the groups of dogs. Histologic appearance of each thyroid gland was scored on the basis of number of follicles and amount of colloid present (normal, increased, or decreased), severity of inflammation and fatty infiltration (none, mild, moderate, or severe), and the presence or absence of mineralization or lipofuscin. The presence of any lesions was recorded.

Each slide was scanned (Aperio ScanScope XT; Leica Biosystems Inc, Buffalo Grove, IL) with a 20X power objective and a camera resolution of 0.4942 microns per pixel. Images were then uploaded (eSlide Manager; Leica Biosystems Inc) as 8-bit JPEG2000-compressed SVS files and visualized with a digital pathology software program (Aperio ImageScope 12.3; Leica Biosystems Inc). After annotation to isolate the thyroid sections, each was manually imported for analysis (Definiens Architect XD 2.7 with Tissue Studio version 4.4.2; Definiens Inc, Cambridge, MA) using an algorithm to segment the tissue into four regions-of-interest (ROIs): colloid, follicular and parafollicular cells, adipose tissue and glass, and RBCs and other stroma. This algorithm was trained on representative input regions in order to classify all the tissue within the ROIs in the final analysis. The program then calculated the total tissue area and the area percentages for each of the ROIs. Additionally, nuclei were detected and scored according to size for each of the ROIs. The analysis output included all quantitative results as well as screen captures of the ROI detection plus overlays for tissue segmentation and cellular scores. For each gland, the average height of the follicular epithelium was measured manually using a digital micrometer for each of 30 follicles selected to include all general regions of the gland.

### Data and statistical analysis

Continuous variables such as clinical pathology findings, serum thyroid hormone and steroid hormone concentrations, urine iodine concentration, and histomorphometry measurements of thyroid tissue, were described as median and range or interquartile range. Individual steroids for which ≤ 20% of values were below the limit of detection (LOD) had missing values imputed by applying a logspline method[45]. For androstenedione, values below the LOD prior to administration of cosyntropin were imputed using a 50% rule method. Steroids for which the majority of dogs had values below the LOD had their missing values excluded from analysis rather than imputed. For measurements of endogenous ACTH, concentrations < 10 pg/ml were assigned a value of zero.

Unmatched numbers of control dogs (n = 30) and dogs with gallbladder mucocele formation (n = 39) underwent thyroid hormone and iodine concentration testing and continuous data were analyzed using unpaired statistical analyses (Kruskal-Wallis One Way ANOVA on Ranks). A reduced subset of breed, sex, and age-matched control dogs (n = 30) and dogs with gallbladder mucocele formation (n = 30) underwent steroid hormone concentration and endogenous ACTH testing and therefore comparisons of continuous data between groups and in response to cosyntropin administration, were analyzed using paired statistical analyses (Wilcoxin signed rank test). To compare the results of thyroid and steroid hormone testing a sample size of 30 dogs per treatment group was chosen based on an estimated ability to demonstrate a 2-fold or greater difference between groups with 80% power and assuming an average coefficient of variance of 0.45 as reported in prior studies from which this variance could be calculated[46, 47].

For diagnostic assays with established reference intervals (clinical pathology, thyroid hormone, TSH and ACTH assay results), those intervals were used to define the percentage of dogs having values outside reference range limits. For ACTH, a reference interval of 10 to 45 pg/ml was used. A laboratory-based diagnosis of hypothyroidism was defined by results demonstrating a low serum total T4 and elevated serum TSH or a low serum FT_4_ as previously proposed[13, 48, 49]. For assays without established reference intervals (i.e. serum steroid concentrations), a 95% double-sided reference interval was calculated from data obtained from control dogs using the Robust method as described in the Clinical and Laboratory Standards Institute (CLSI) Guidelines EP28-A3c and recommended by the American Society of Veterinary Clinical Pathology for sample sizes ranging from ≥ 20 to < 40 [50]. Data were tested for outliers using the method based on Reed et al [51]. Ninety percent confidence intervals for the reference limits were estimated using bootstrapping (percentile interval method[52]). Statistical analyses were performed using MedCalc for Windows, version 18.11 (MedCalc Software, Ostend, Belgium). In the control group of dogs, there were no significant differences between spayed female and neutered male dogs in the concentrations of steroid hormones before or after exogenous administration of ACTH. Accordingly, the control values of each steroid from female and male dogs were combined to create a control reference interval for each steroid. Based on these reference intervals, the % of dogs having steroid concentrations outside the reference interval was calculated. Differences in the % of control dogs and dogs with gallbladder mucocele formation having values outside of reference range limits were tested for significance using Chi Square and Fisher Exact Tests. Statistically significant results were reported as odds ratio, 95% confidence interval, and p-value.

A Pearson product-moment correlation coefficient (r) was computed to assess strength of the linear relationship between the following continuous variables (age, cholesterol, alkaline phosphatase activity (ALP), gamma-glutamyl transferase activity (GGT), total bilirubin, blood urea nitrogen (BUN), creatinine, lipase, amylase, TT3, TT4, FT3, FT4, TSH, UICR, post-cosyntropin cortisol, and endogenous ACTH). The correlation coefficient can be generally interpreted to reflect a weak (r<0.4), moderate (r>0.4 to 0.7), or strong relationship (r>0.7) between the variables and with the positive or negative value of r reflecting the direction of the association[53]. All statistical analyses were performed using commercially available software (Sigma Plot12, Systat Software, Inc. San Jose, CA and Prism version 7.03, GraphPad Software, La Jolla, CA).

Statistical results of experimental data undergoing multiple testing underwent a Benjamini-Hochberg procedure[54] using a false-discovery rate of 0.15. All results reported as statistically significant had a p-value of <0.05 and Benjamini-Hochberg corrected p-value <0.15.

## Results

### Description of case dogs

Ninety-seven dogs, suspected to have gallbladder mucocele formation, were considered for inclusion in the study. Thirty-nine dogs (40%) met the inclusion criteria. The remaining 58 (60%) dogs had one or more exclusion criteria. Twenty five dogs were excluded based on failure to confirm gallbladder mucocele formation upon review of ultrasonographic images by an ACVR-boarded radiologist (G.S.) or at the time of surgery or post-mortem examination. Twelve dogs were receiving medications known or suspected to interfere with thyroid function testing including exogenous glucocorticoids, sulfa-containing drugs, or non-steroidal anti-inflammatory drugs. The remaining dogs were assessed to be too medically unstable to participate in the study (n = 9), were reproductively intact (n = 5), were documented to have an historical diagnosis of hypothyroidism or were receiving levothyroxine (n = 6), or were previously diagnosed with hyperadrenocorticism (n = 1).

Dogs with gallbladder mucocele formation that were enrolled into the study were represented by 19 breeds including 11 Shetland Sheepdogs, 4 American Cocker Spaniels, 3 Beagles, 2 Bichon Frise, 2 Chihuahuas, 2 Miniature Poodles, 2 Pugs, 2 Mixed Breed Dogs, and 1 each of the following breeds: American Staffordshire Terrier, Border Collie, Border Terrier, Cavalier King Charles Spaniel, Fox Terrier, Kerry Blue Terrier, Labrador Retriever, Miniature Schnauzer, Pomeranian, Shih Tzu, and West Highland White Terrier. Ages of the affected dogs ranged from 2 to 16 years (median, 10 years). There were 22 castrated males and 17 spayed females and the median body weight was 9.0 kg (range 5.0 to 34.2 kg). The non-thyroidal illness severity scores of the dogs at the time of participation in the study were as follows: 0 (absent) in 14 (35.9%) dogs, 1 (mild) in 8 (20.5%) dogs, 2 (moderate) in 8 (20.5%) dogs, and 3 (severe) in 9 (23.1%) dogs.

### Description of control dogs

Thirty-eight dogs of similar age, predisposed breed, and spayed or castrated were screened for inclusion in the study as controls. Thirty dogs met the inclusion criteria. All 8 dogs that were excluded had an abnormal ultrasonographic appearance of the gallbladder. The 30 control dogs enrolled in the study represented 16 breeds including 11 Shetland Sheepdogs, 3 American Cocker Spaniels, 2 Chihuahuas, 2 Bichon Frise, and 1 each of the following breeds: American Staffordshire Terrier, Beagle, Border Collie, Border Terrier, Cavalier King Charles Spaniel, Fox Terrier, Kerry Blue Terrier, Miniature Schnauzer, Shih Tzu, Pug, Miniature Poodle, and Labrador Retriever. Ages of the control dogs ranged from 6 to 13 years (median, 10 years). There were 16 castrated males and 14 spayed females and the median body weight was 9.5 kg (range 2.7 to 35.6 kg). All control dogs had an illness severity score of 0 (absent). There was not a statistically significant difference in age, sex, or body weight between control dogs and dogs with gallbladder mucocele formation.

### Diagnosis of gallbladder mucocele formation

All 39 dogs had gallbladder mucocele formation diagnosed by means of ultrasonography of the gallbladder. Indications for the ultrasound examination in these dogs included routine screening for gallbladder mucocele formation in an otherwise healthy dog (10 dogs); clinical signs of gastrointestinal illness, increased liver enzyme activities and/or increased serum total bilirubin (21 dogs); further evaluation of urinary tract disease (urolithiasis, stranguria, azotemia)(4 dogs); investigation of inappetence, weakness or syncope in a cardiology patient (3 dogs); and as a survey for metastatic disease (1 dog). In 14/39 (36%) dogs, gallbladder mucocele formation was additionally confirmed by histopathology of gallbladder tissue obtained at the time of surgery (10 dogs) or at post-mortem examination (4 dogs). All 30 control dogs had no evidence if mucocele formation on gallbladder ultrasound examination. In all control dogs, indication for ultrasound examination was for the purpose of screening for inclusion in this study.

### Clinicopathologic findings

Results of CBC and a serum biochemistry profile were obtained for 38/39 (97.4%) dogs with gallbladder mucocele formation and all 30 control dogs. Compared to control dogs, dogs with gallbladder mucocele formation had significantly more polymorphonuclear leukocytes and bands and higher activities for liver enzymes (alkaline phosphatase, ALT, and GGT), lipase, and amylase. Also observed was a higher serum total bilirubin and cholesterol and lower serum albumin in dogs with gallbladder mucocele formation compared to control dogs (Table 1).

**Table 1:** Selected complete blood cell count and serum biochemical analysis findings in 38^§^ dogs with gallbladder mucocele formation and 30 control dogs that fit inclusion criteria for this study.

Clinical Pathological Variable	No Gallbladder Mucocele (30 dogs)			Gallbladder Mucocele (38 dogs^§^)			Reference range	Chi-square Odds Ratio		
	Median	Range	Number (%) of dogs with abnormal value	Median	Range	Number (%) of dogs with abnormal value		Odds Ratio	95% CI	*p*-value
**Complete blood cell count**										
Packed cell volume (%)	44	30–52	3 (10)	41	22–61	13 (35)*	39–58	4.7	1.2–18.4	**0.04**
Plasma protein (g/dl)	7.2	5.8–8.3	12 (43)	7.3	4.3–10.0	13 (42)	6.1–7.5	0.78	0.29–2.1	0.812
Total white blood cells (× 10^3^/μl)	7.545	3.460–13.730	5 (17)	10.670***	4.670–66.330	18 (49)*	4.39–11.61	4.5	1.4–14.2	**0.016**
Polymorphonuclear leukocytes (× 10^3^/μl)	5.460	2.214–10.450	6 (20)	8.610***	3.468–55.054	16 (43)	2.841–9.112	2.9	0.97–8.7	0.094
Bands (× 10^3^/μl)	0.0	0.0–0.210	10 (33)	0.217***	0–6.568	25 (68)*	0.0–0.0	3.8	1.4–10.6	**0.016**
Platelets (× 10^3^/μl)	353	189–616	4 (13)	381	73–820	8 (22)	191–468	1.7	0.47–10.6	0.016
**Serum biochemical analysis**										
Alkaline phosphatase (IU/L)	52	6–251	5 (17)	308***	21–5236	27 (71)***	16–140	12.3	3.74–40.3	**<0.001**
ALT (IU/L)	48	11–215	10 (33)	172***	11–5393	27 (71)**	12–54	4.9	1.75–13.8	**0.004**
GGT (IU/L)	0.0	0–6	0 (0)	12***	0–112	21 (55)***	0–6	—	—	**<0.001†**
Total bilirubin (mg/dl)	0.0	0.0–0.1	0 (0.0)	0.1***	0.0–11.5	12 (32)**	0–0.2	—	—	**0.002†**
Cholesterol (mg/dl)	259	165–452	5 (17)	323*	107–754	18 (47)*	124–344	4.5	1.42–12.2	**0.016**
Blood urea nitrogen (mg/dl)	16	8–36	2 (7)	17	5–179	12 (32)*	8–26	6.5	1.32–31.7	**0.026**
Creatinine (mg/dl)	0.8	0.5–1.1	0 (0.0)	0.75	0.2–5.9	4 (10)	0.7–1.5	—	—	0.124**†**
Albumin (g/dl)	3.6	2.9–4.6	5 (17)	3.3**	1.6–4.2	13 (34)	3–3.9	2.6	0.81–8.4	0.117
Lipase (IU/L)	83	24–1032	3 (10)	144***	25–3920	19 (50)**	12–147	9.0	2.3–34.8	**0.001**
Amylase (IU/L)	664	67–1496	1 (3)	825*	363–3143	11 (29)*	236–1337	11.8	1.4–97.8	**0.015**

Comparison of median values performed using Kruskal-Wallis One Way ANOVA on Ranks. Comparison of proportions performed using Chi-square statistic.

*p<0.05

**p<0.01, and

***p<0.001 (Benjamini-Hochberg FDR ≤ 0.15).

†P-value represents Fisher-Exact test probability.

^§^ Testing was not performed in 1 dog with gallbladder mucocele formation

ALT, alanine aminotransferase; GGT, gamma-glutamyl transferase

### Steroid hormone assay results

Among the 17 unique steroids included in the utilized assay, 8 steroids were present in > 90% of all dogs at serum concentrations greater than the LOD before or after the administration of synthetic cosyntropin. For these steroids, missing values were imputed, and 95% reference intervals were constructed from the control dog data. Considering this subset of steroids, no significant differences were observed between control and gallbladder mucocele groups in the number of dogs having ≥ 1 steroid hormone (or any individual steroid hormone) above of the reference interval before or after the administration of cosyntropin (S1 and S2 Tables).

**Fig 1:**
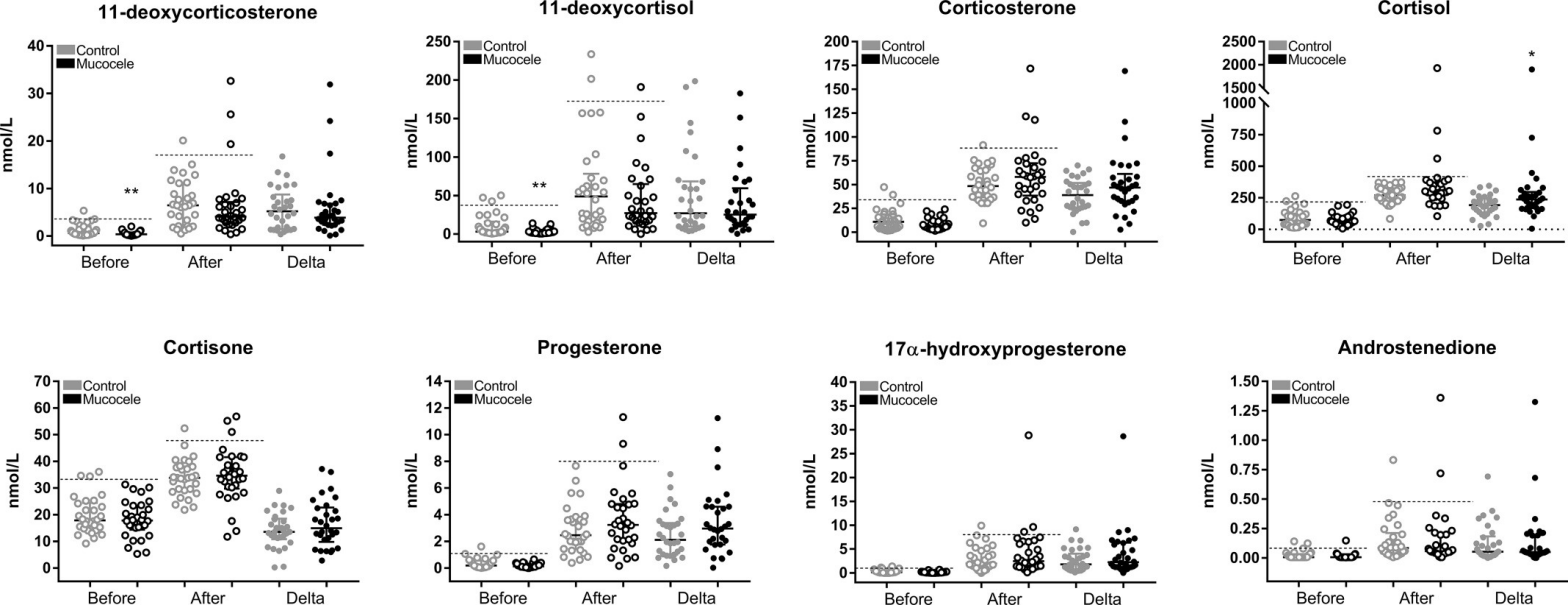
Measurements of serum steroid concentrations before and 1 hour after administration of cosyntropin to control dogs and dogs diagnosed with gallbladder mucocele formation. Data points represent individual dogs. Before and after refer to concentrations measured prior to and following administration of cosyntropin, respectively. Delta refers to the absolute change in steroid concentration after cosyntropin administration (after–before). Horizontal dashed lines indicate the upper limit of the reference interval established by CLSI C28-A3 Robust method using concentrations measured in control dogs in this study. Confidence interval (90%) for upper limit reference interval in control dogs is reported in S1 and S2 Tables. Bars represent median and interquartile range. *P<0.05, **P <0.01 Wilcoxin signed rank test compared to control dogs under same conditions (Benjamini-Hochberg FDR ≤ 0.15).

Before administration of cosyntropin, dogs with gallbladder mucocele formation had significantly lower median serum concentrations of 11-deoxycorticosterone and 11-deoxycortisol compared to control dogs. For both groups of dogs, a significant increase in the median concentration of each of the 8 steroids was observed after administration of cosyntropin. After administration of cosyntropin, dogs with gallbladder mucocele formation had a greater magnitude of increase in both corticosterone and cortisol compared to control dogs, although only cortisol remained significant after adjusting for multiple testing (Fig 1). Data relating cortisol concentrations before and after administration of cosyntropin in individual dogs is shown in S1 Fig.

**Fig 2:**
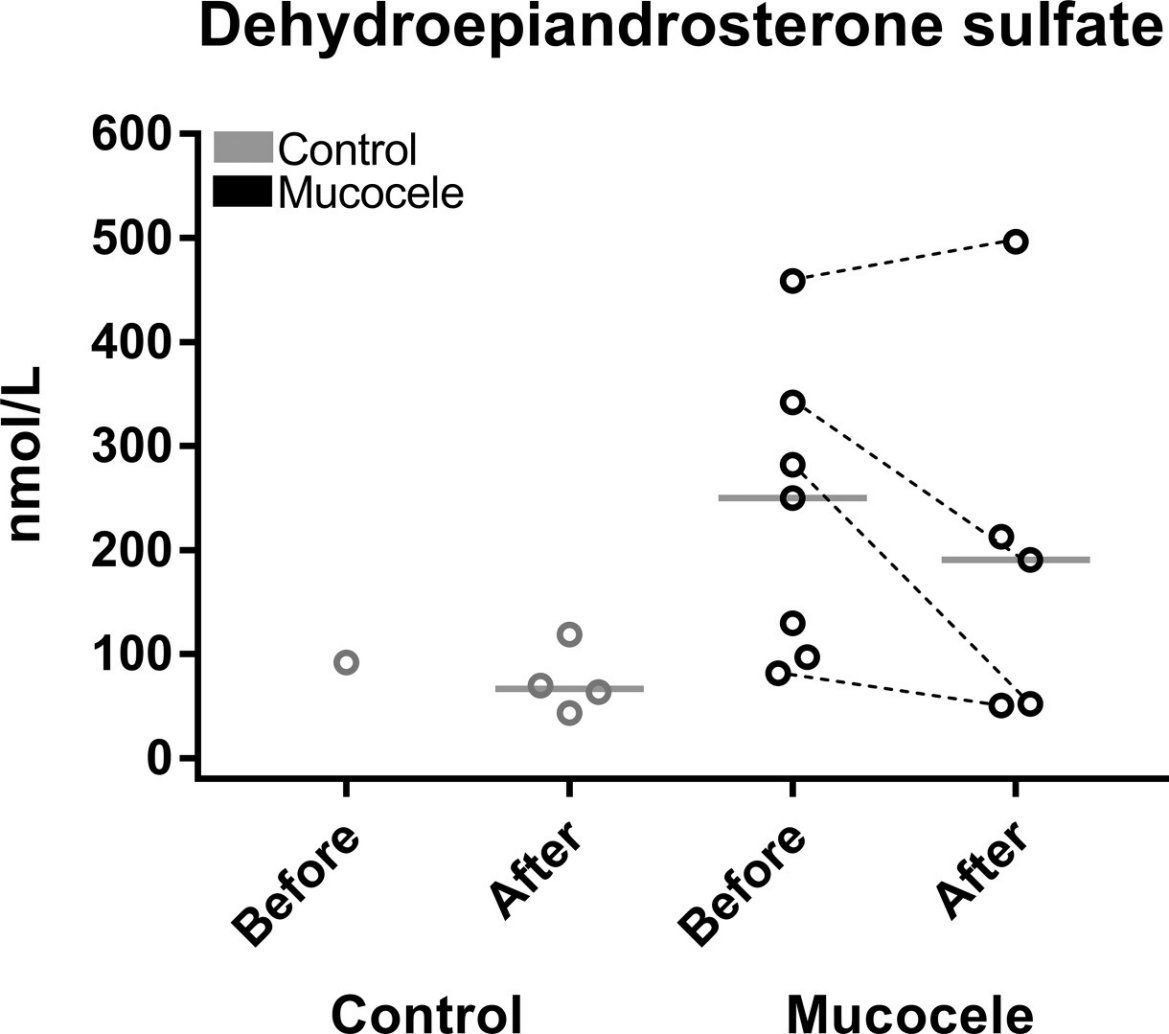
Measurements of serum dehydroepiandosterone sulphate (DHEAS) concentrations before and 1 hour after administration of cosyntropin to control dogs and dogs diagnosed with gallbladder mucocele formation. Data points represent individual dogs with concentrations above the lower limit of detection. Before and after refer to concentrations measured prior to and following administration of cosyntropin, respectively. Dashed lines indicate dogs for which a measurable concentration of DHEAS was obtained both before and after administration of cosyntropin. Bars represent median. *P<0.05 Kruskal-Wallis One Way Analysis of Variance on Ranks compared to control dogs after administration of cosyntropin (Benjamini-Hochberg FDR ≤ 0.15).

Nine of the 17 assayed steroids were present at concentrations greater than LOD in only a subset of dogs (Table 2). Considering data for only those dogs with values greater than LOD, a significantly greater number of dogs with gallbladder mucocele formation (7/30, 23%) had measurable concentrations of dehydroepiandrosterone sulfate (DHEAS) compared to control dogs (1/30, 3%) before administration of cosyntropin (Fig 2). The median concentration of DHEAS before cosyntropin in dogs with gallbladder mucocele formation was significantly greater than for control dogs after cosyntropin administration. No dogs with gallbladder mucocele formation had detectable concentrations of aldosterone prior to administration of cosyntropin, but mucocele dogs had significantly higher concentrations of aldosterone than control dogs after administration of cosyntropin (Table 2). Infrequent detection of dihydrotestosterone, androsterone, estrone, and etiocholanolone was anticipated in these spayed and castrated dogs.

**Table 2:** Serum concentrations (in nmol/L) of 9 steroid hormones measured at concentrations > LOD in less than 50% of dogs before and 1 hour after intravenous administration of synthetic cosyntropin.

Steroid	Number (%) of dogs with steroid > LOD				Median (IQR) steroid concentration in nmol/L			
	Control (n = 30)		Mucocele (n = 30)		Control (n = 30)		Mucocele (n = 30)	
	Before	After	Before	After	Before	After	Before	After
Testosterone	3 (10)	15 (50)	2 (7)	12 (40)	0.0211 (0.0153–0.0218)	0.0350 (0.0177–0.0558)	0.0340 (0.0211–0.0468)	0.0274 (0.0191–0.0414)
Aldosterone	4 (13)	11 (37)	0 (0)*	8 (27)	0.298 (0.135–0.383)	0.168 (0.125–0.280)	ND	**0.376 (0.210–0.487)§**
Dehydroepiandrosterone sulfate	1 (3)	4 (13)	7 (23)*	5 (17)	91.7 (1 dog)	66.6 (48.2–106.4)	**249.9 (96.9–342)§†**	190.5 (51.3–354.7)
Dihydrotestosterone‡	3 (10)	1 (3)	3 (10)	3 (10)	0.580 (0.0296–0.869)	0.577 (1 dog)	0.222 (0.164–0.694)	0.197 (0.149–0.639)
Androsterone‡	1 (3)	0 (0)	1 (3)	3 (10)	0.0258 (1 dog)	ND	0.0296 (1 dog)	0.0279 (0.0258–0.0665)
Estrone‡	3 (10)	0 (0)	0 (0)	0 (0)	0.0233 (0.0215–0.0270)	ND	ND	ND
Estradiol‡	0 (0)	0 (0)	1 (3)	0 (0)	ND	ND	0.031 (1 dog)	—
Etiocholanolone‡	0 (0)	1 (3)	0 (0)	0 (0)	ND	0.481 (1 dog)	ND	ND
Dehydroepiandrosterone‡	0 (0)	0 (0)	0 (0)	0 (0)	ND	ND	ND	ND

*P<0.05 Fisher Exact Test compared to control dogs before cosyntropin

^§^P<0.05 Kruskal-Wallis One Way Analysis of Variance on Ranks (Benjamini-Hochberg FDR ≤ 0.15).

†Significantly greater compared to after- cosyntropin concentration in control dogs

‡Steroids for which ≤ 3 dogs had concentrations > LOD did not undergo statistical analysis

ND, not detected at concentrations >LOD in any dogs

**Fig 3:**
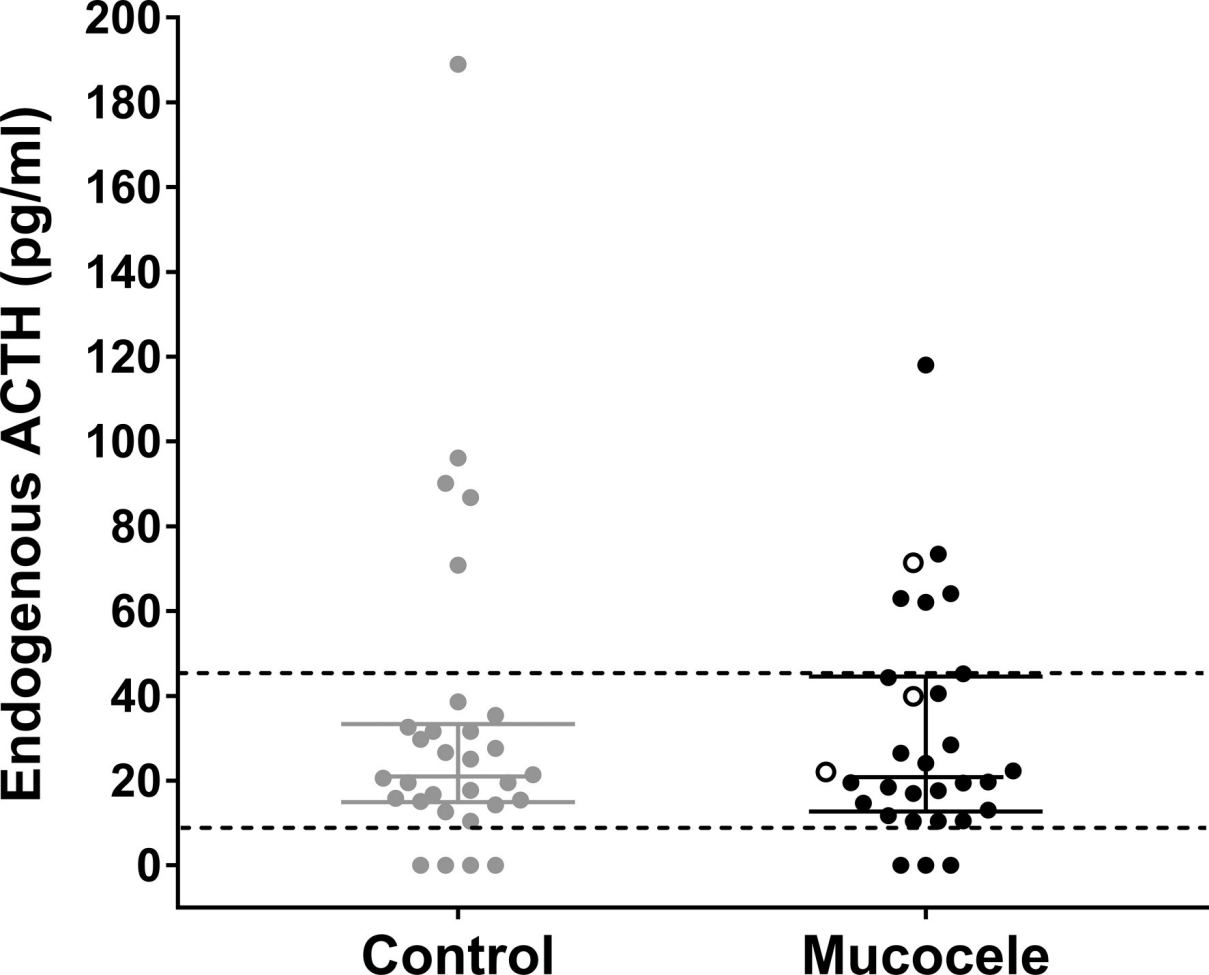
Plasma ACTH concentration in control dogs and dogs with gallbladder mucocele formation. Data represent individual dogs with reference range limits shown as dashed lines. Bars represent median and interquartile range. Open circles represent dogs with gallbladder mucocele formation that had concurrent post-cosyntropin serum cortisol concentrations above the calculated reference range (as shown in Fig 1).

Median pre-cosyntropin plasma endogenous ACTH concentration did not differ between control dogs and dogs with gallbladder mucocele formation. Similar numbers of control and gallbladder mucocele dogs had concentrations above and below the reference interval (Fig 3). There was no significant correlation between ACTH and concentrations of 11-deoxycorticosterone, 11-deoxycortisol, corticosterone, cortisol, cortisone, progesterone, 17α-hydroxyprogesterone, or androstenedione among either control dogs or dogs with gallbladder mucocele formation (data not shown).

### Thyroid hormone assay results

**Fig 4:**
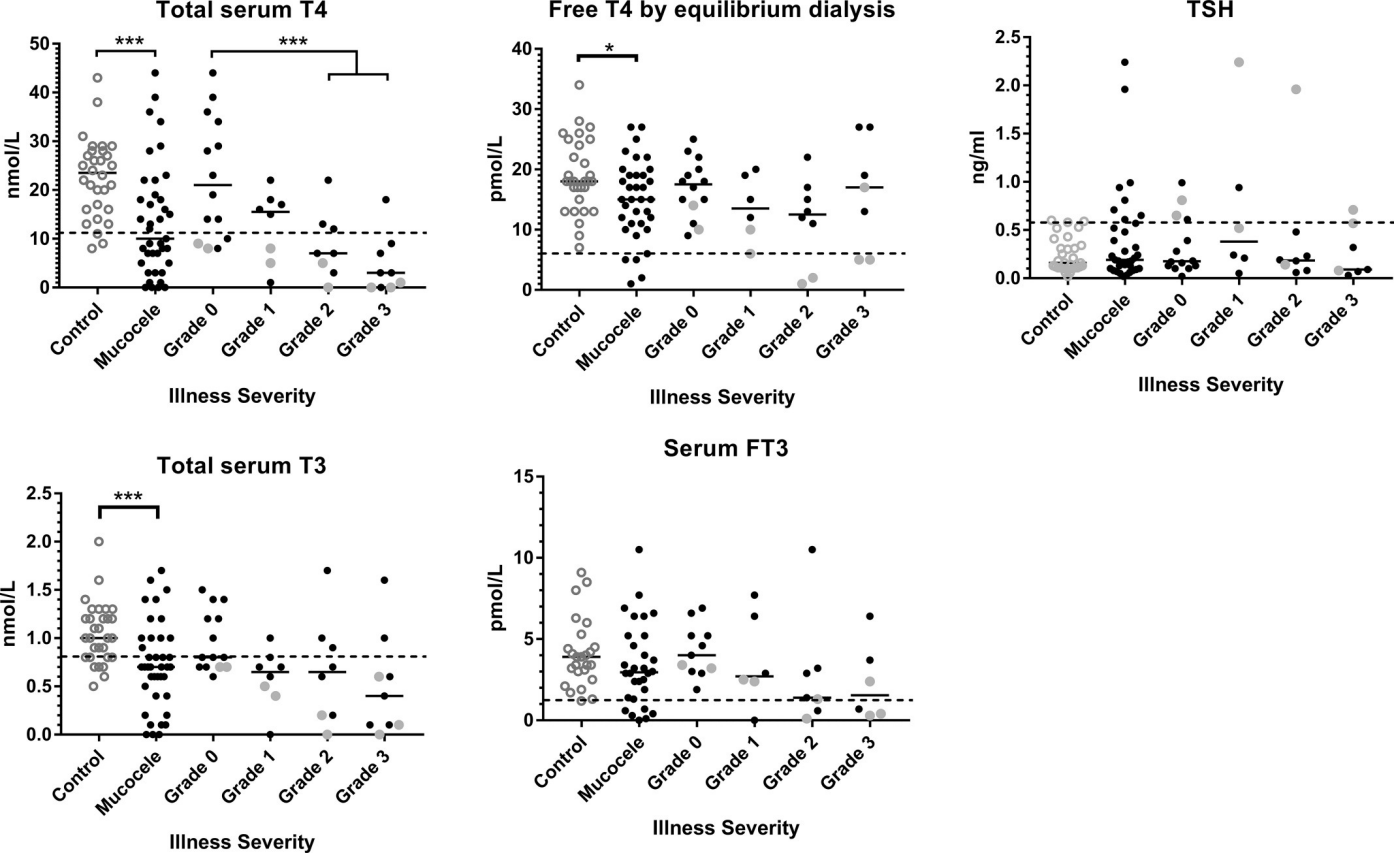
Thyroid hormone assay results obtained from control dogs and dogs diagnosed with gallbladder mucocele formation. For dogs with gallbladder mucocele formation, values are shown subdivided by non-thyroidal illness severity score. The horizontal dashed line designates lower limit of the laboratory reference range (total T4, FT4, total T3, and FT3) and upper limit of the laboratory reference range (TSH). Among dogs with gallbladder mucocele formation, solid gray data points represent dogs meeting proposed criteria for diagnosis of hypothyroidism. Bar = median. *p<0.05 and ***p<0.001 Kruskal Wallis One Way ANOVA on Ranks (Benjamini-Hochberg FDR ≤ 0.15). Significant differences between illness severity groups, if present, determined by post-hoc Dunn’s test.

Sixty-nine percent (27/39) of dogs with gallbladder mucocele formation had at least one measurement out of reference range on the thyroid profile compared to 37% (11/30) of control dogs (OR 3.9, 95% CI 1.4–10.6; p = 0.014). Summary data for each assay for both groups of dogs are shown in Table 3 and presented individually in Fig 4.

**Table 3:** Thyroid hormone, thyroid hormone stimulating hormone, and autoantibody results in 39 dogs with gallbladder mucocele formation and 30 control dogs meeting the inclusion criteria for this study.

Thyroid Profile Variables	No Gallbladder Mucocele				Gallbladder Mucocele				Reference range	Chi-square Odds Ratio		
	Median	Range	Number (%) of dogs with value out of reference range		Median	Range	Number (%) of dogs with value out of reference range					
			Below	Above			Below	Above		Odds Ratio	95% CI	*p*-value
Total T4 (nmol/L)	23.5	8–43	2/30 (7)	0/30 (0)	10***	0.0–44	20/39 (51)***	0/39 (0)	11–60	14.7	3.1–70.5	**<0.001**
Total T3 (nmol/L)	1.0	0.5–2.0	5/30 (17)	0/30 (0)	0.7***	0.0–1.7	23/39 (59)***	0/39 (0)	0.8–2.1	7.2	2.3–22.8	**<0.001**
Free T4 by dialysis (pmol/L)	18.9	7.0–34.0	0/30 (0)	0/30 (0)	15.0*	1.0–27.0	5/35 (14)	0/35 (0)	6–42	—	—	0.057†
Free T3 (pmol/L)	3.9	1.2–9.1	0/25 (0)	2/25 (8)	2.9	0.0–10.5	6/30 (20)*	1^§^/30 (3)	1.2–8.2	—	—	**0.027**†
Thyroid Stimulating Hormone (ng/ml)	0.16	0.02–0.6	0/30 (0)	2/30 (7)	0.19	0.02–2.24	0/35 (0)	8/35 (23)	0.00–0.58	4.1	0.81–21.3	0.092
T4 Autoantibody (%)	10.0	0.0–12.0	0/25 (0)	0/25 (0)	7.0*	0.0–12.0	0/32 (0)	0/32 (0)	0–20	NA	NA	NA
T3 Autoantibody (%)	4.0	0.0–7.0	0/28 (0)	0/28 (0)	5.0*	0.0–28.0	0/34 (0)	2/34 (6)	0–10	—	—	0.497†
Thyroglobulin Autoantibody (%)	7.0	0.0–77.0	0/30 (0)	2/30 (7)	6.0	0.0–19.0	0/35 (0)	0/35 (0)	0–35	—	—	0.209†

Comparison of median values performed using Kruskal-Wallis One Way ANOVA on Ranks.

Comparison of proportions performed using Chi-square statistic.

*p<0.05

**p<0.01, and

***p<0.001 (Benjamini-Hochberg FDR ≤ 0.10).

†P-value represents Fisher-Exact test probability.

§ This dog did not have identified autoantibodies.

There was a significantly lower median serum concentration of total T4 in dogs with gallbladder mucocele formation compared to control dogs with 51% (20/39) of gallbladder mucocele dogs and 7% (2/30) of control dogs having a value below the reference range. Compared to control dogs, dogs with gallbladder mucocele formation were 14.7 times more likely to have a serum total T4 concentration below reference range limits. There was a significantly lower median serum concentration of total T3 in dogs with gallbladder mucocele formation compared to control dogs with 59% (23/39) of gallbladder mucocele dogs and 17% (5/30) of control dogs having a value below the reference range. Compared to control dogs, dogs with gallbladder mucocele formation were 7.2 times more likely to have a serum total T3 concentration below reference range limits (Fig 4). Total T4, T3, or both were not detected in the serum of 6/39 (15%) dogs with gallbladder mucocele formation. Both total T4 and total T3 were below reference range limits in 19/39 (49%) dogs, followed by low total T3 alone in 4/39 (10%) or low total T4 alone in 2/39 (5%) dogs with gallbladder mucocele formation.

There was a significantly lower median serum concentration of FT_4_ in dogs with gallbladder mucocele formation compared to control dogs with 14% (5/35) of gallbladder mucocele dogs and 0% (0/30) of control dogs having a value below the reference range. Free T_4_ was below the limit of detection in the serum of one dog with gallbladder mucocele formation. The median serum concentration of FT_3_ was not significantly different between dogs with gallbladder mucocele formation and control dogs, however a significantly greater percentage of dogs with gallbladder mucocele formation had a serum concentration of FT_3_ below reference range (6/30; 20%) compared to control dogs (0/25; 0%). An increase in serum concentration of TSH above reference range limits was observed in 23% (8/35) of dogs with gallbladder mucocele formation and 7% (2/30) of control dogs.

An increase above reference range in serum percent TgAA was observed in 2/34 (7%) control dogs and 0/30 (0%) dogs with gallbladder mucocele formation. Two dogs with gallbladder mucocele formation (a Shetland sheepdog and a Border Collie) had a T3AA value above the reference range. No dog in the study had a T4AA value above the reference range.

Twenty-six percent (9/35) of dogs with gallbladder mucocele formation met diagnostic criteria for diagnosis of hypothyroidism on the basis of having a low serum total T4 and elevated serum TSH or a low serum FT_4_ [13, 48, 49]. Among the 9 dogs meeting the diagnostic criteria, 5 dogs had all 3 abnormalities present. Four dogs with gallbladder mucocele formation were not included in the analysis due to a missing FT_4_ or TSH measurement. None of the control dogs met diagnostic criteria for diagnosis of hypothyroidism.

### Urine inorganic iodine and iodine:Creatinine ratio

**Fig 5:**
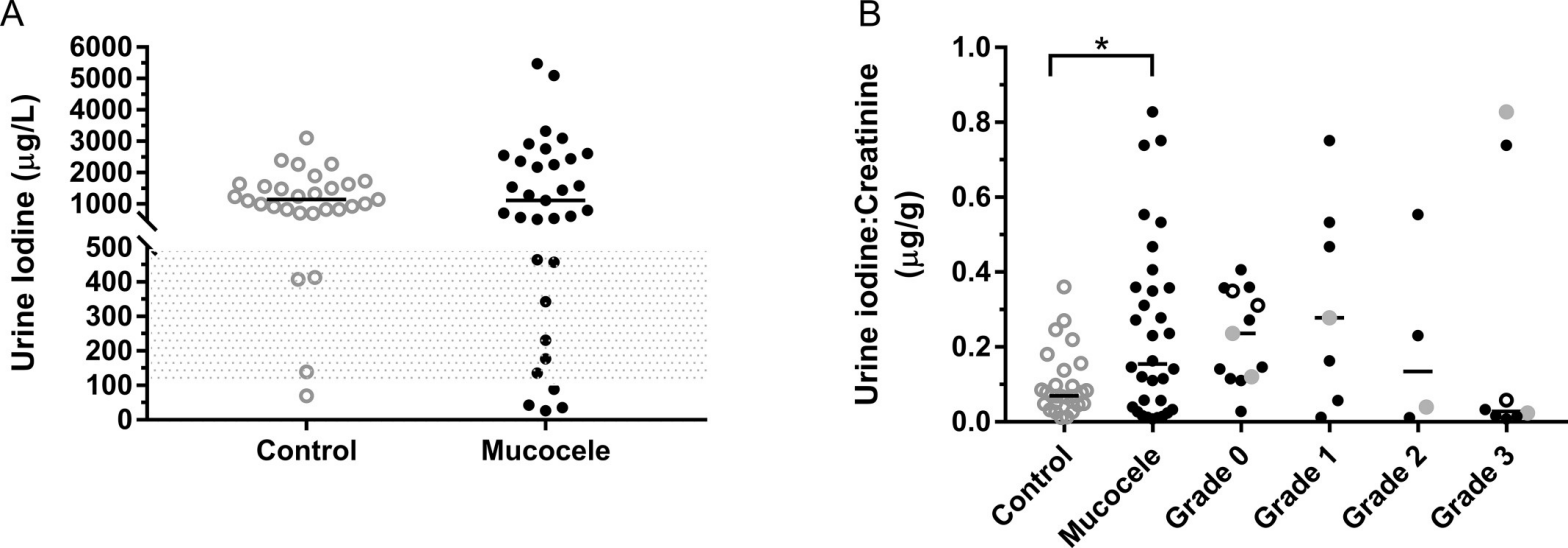
**Urine iodine concentration (A) and urine iodine to creatinine ratio (UICR) (B) of control dogs and dogs diagnosed with gallbladder mucocele formation.** Panel A demonstrates values for urine iodine concentration in individual dogs. Bar = median. Shaded area represents the NRC recommended daily intake of iodine (in μg) as expressed on the basis of metabolic body weight. Panel B demonstrates UICR for both groups of dogs and in dogs with gallbladder mucocele formation as subdivided by illness severity score. Among dogs with gallbladder mucocele formation, filled gray data points represent dogs meeting proposed criteria for diagnosis of hypothyroidism. Open circle data points represent 3 dogs that were subsequently euthanized and had thyroid tissue obtained for iodine measurement. Bars = median. *p<0.05 Kruskal Wallis One Way ANOVA on Ranks.

Inorganic iodine and creatinine were measured in the urine of 32 dogs with gallbladder mucocele formation and 29 control dogs. Both groups of dogs demonstrated a high median and wide range in concentration of urine iodine that did not differ significantly between control dogs and dogs with gallbladder mucocele formation (Fig 5A). Based on the National Research Council recommended daily allowance for iodine intake in the dog, and as expressed on the basis of metabolic body weight (29.6 μg/kg^0.75^)[55, 56], commercial diets should provide iodine within a minimum range of 100 to 500 μg per day. Assuming a conservative urine output of 30 ml/kg/day, dogs in this study can be estimated to have excreted anywhere from 6 μg to more than 3,324 μg of iodine in excess of their daily thyroid requirement. Dogs with gallbladder mucocele formation had a significantly higher median urine iodine:creatinine ratio (UICR; μg/g) of 0.154 (interquartile range (IQR), 0.0346 to 0.359) compared to control dogs (UICR = 0.0692; IQR, 0.0482 to 0.118)(p = 0.048, Kruskal Wallis One Way ANOVA on Ranks) (Fig 5B). There was no significant association between non-thyroidal illness severity score and urine iodine:creatinine ratio in dogs with gallbladder mucocele formation.

### Influence of illness severity

A significant impact of non-thyroidal illness severity score on median measurements of cortisol and thyroid hormones, endogenous ACTH and TSH, and UICR was only observed for serum total T4. Dogs with an illness severity score of 2 or 3 (moderate to severe) were more likely to have a serum total T4 below the reference range compared to dogs with an illness severity score of 0 or 1 (absent to mild)(OR 6.0, 95% CI 1.6–29.3; p = 0.015). There was no significant association between an illness severity score of 2 to 3 versus 0 to 1 and presence of an out-of-reference-range test result for total T3, FT_4_, FT_3_, TSH, post-cosyntropin cortisol, endogenous ACTH, or having met the diagnostic criteria for hypothyroidism or hyperadrenocorticism (data not shown).

### Correlations between serum biochemistry, post-cosyntropin cortisol, and thyroid hormone concentrations

Correlations were performed to provide insight into the pathogenesis or confounding influence of serum biochemistry abnormalities and their relationship to the results of simultaneously measured post-cosyntropin cortisol and thyroid hormone concentrations in dogs with gallbladder mucocele formation.

Serum cholesterol was positively correlated with serum concentration of total T3 and FT_3_ in dogs with gallbladder mucocele formation (TT3; r = 0.436, n = 38, p = 0.006 and FT_3_; r = 0.506, n = 29, p = 0.005). For both groups of dogs, there was no significant correlation between serum cholesterol and post-cosyntropin cortisol or endogenous ACTH concentration. Dogs having gallbladder mucocele formation and a serum cholesterol above reference range limits were no more likely than dogs with normal serum cholesterol to have met the defined criteria for diagnosis of hypothyroidism or hyperadrenocorticism. In dogs with gallbladder mucocele formation, serum cholesterol was positively correlated with biochemical indices of cholestasis, ALP (r = 0.394, n = 38, p = 0.014) and total bilirubin (r = 0.406, n = 38, p = 0.01).

There were no significant correlations between serum biochemistry values and post-cosyntropin cortisol or endogenous ACTH concentrations in dogs with gallbladder mucocele formation other than a positive correlation between GGT activity and post-cosyntropin cortisol (r = 0.449, n = 30, p = 0.013). In control dogs, ALP was positively correlated with post-cosyntropin cortisol concentration (r = 0.484, n = 28, p = 0.009).

Serum albumin was positively correlated with serum concentration of total T4 (r = 0.466, n = 30, p = 0.009) and total T3 (r = 0.575, n = 30, p = 0.0009) in control dogs and with total T4 (r = 0.525, n = 38, p = 0.0007), total T3 (r = 0.542, n = 38, p = 0.0004), and FT_3_ (r = 0.596, n = 29, p = 0.0006) in dogs with gallbladder mucocele formation. Dogs with gallbladder mucocele formation that had serum albumin concentration below reference range limits were no more likely to have abnormal concentrations of thyroid hormones, TSH, post-cosyntropin cortisol, or endogenous ACTH compared to dogs with gallbladder mucocele formation and normal serum albumin. In dogs with gallbladder mucocele formation, serum albumin was negatively correlated with BUN (r = -0.414, n = 38, p = 0.0097), and creatinine (r = -0.426, n = 38, p = 0.0077) concentration.

Among all dogs with gallbladder mucocele formation, there were moderate correlations between increasing age and decreasing FT_4_ concentration (r = -0.450, n = 35, p = 0.007), increasing TSH concentration (r = 0.492, n = 35, p = 0.003), and increasing UICR (r = 0.426, n = 32, p = 0.015). Significant correlations between age and FT_4_, TSH, and UICR in control dogs was not observed. In dogs with gallbladder mucocele formation, UICR was positively correlated with post-cosyntropin cortisol concentration (r = 0.654, n = 24, p = 0.0005) and serum amylase activity (r = 0.454, n = 31, p = 0.01).

In control dogs, ACTH was positively correlated with TSH (r = 0.497, n = 28, p = 0.007). In dogs with gallbladder mucocele formation, there was no correlation between post-cosyntropin cortisol concentration or ACTH with any individual serum thyroid hormone measurement (total T4, total T3, FT_4_ or FT_3_), or TSH. Dogs with gallbladder mucocele formation and having a post-cosyntropin cortisol concentration above the calculated reference range were no more likely than other dogs to have met the criteria for diagnosis of hypothyroidism. Correlation matrices between serum biochemistry results and results of serum thyroid hormone testing, TSH, post-cosyntropin cortisol, ACTH, and UICR are shown for control dogs (S3 Table) and dogs with gallbladder mucocele formation (S4 Table).

### Measurement of organic iodine in thyroid tissue from euthanized dogs

Recognition of significantly increased UICR in dogs with gallbladder mucocele formation prompted interest in thyroid gland content of organic iodine, which requires the collection of fresh frozen thyroid tissue. Accordingly, thyroid lobes were prospectively collected from 10 dogs with gallbladder mucocele formation immediately following euthanasia. Only 3 of these dogs had undergone thyroid hormone assay testing prior to euthanasia. In each case, gallbladder mucocele formation was confirmed grossly and histologically post-mortem. Breeds represented included Shetland sheepdogs (n = 3), Border Collie, Border Terrier, Chihuahua, Lhasa Apso, Miniature Schnauzer, Shih Tzu, and West Highland White Terrier (n = 1 each). Dogs ranged in age from 10 to 17 years (median, 12.5 years). Control thyroid gland tissue was obtained from research Foxhounds aged 4 years (n = 4), mixed-breed shelter dogs ranging in age from 2 years to geriatric (n = 5), a 14 year old Chihuahua, and a 5 year old Shetland sheepdog. All control dogs underwent euthanasia over the same time interval as dogs with gallbladder mucocele formation and absence of gallbladder mucocele formation was confirmed by gross inspection of the gallbladder at the time of post-mortem examination.

**Fig 6:**
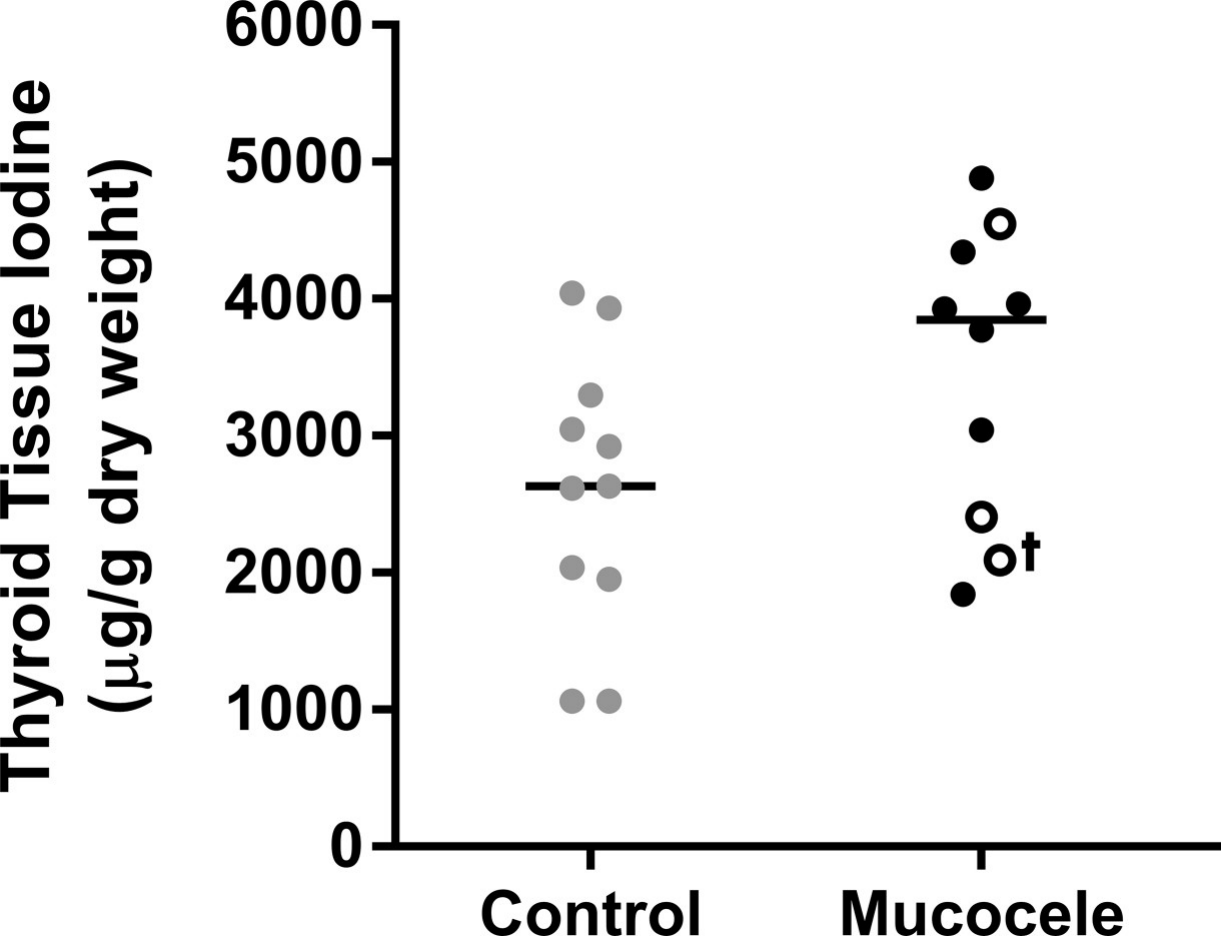
Thyroid iodine content of control dogs and dogs diagnosed with gallbladder mucocele formation. Among dogs with gallbladder mucocele formation, open circle data points represent 3 dogs that had concurrent thyroid hormone assay testing. † serum total T4 and total T3 were below reference range. Bars = median.

Thyroid glands from dogs with gallbladder mucocele formation had a non-significantly greater concentration of organic iodine (mean ± SD, 3,482 ± 1,071 μg/g dry weight) than glands from control dogs (2,600 ± 1,006)(p = 0.067, one-way ANOVA)(Fig 6). For the 3 dogs with gallbladder mucocele formation that also had thyroid hormone testing and UICR measured, 2 dogs had normal thyroid hormone test results and 1 dog had low serum total T4 and total T3. UICR results for these 3 dogs can be discerned from Fig 5B.

### Retrospective examination of archival thyroid tissue from dogs with gallbladder mucocele formation

Twenty-two dogs with a histologic diagnosis of gallbladder mucocele formation and undergoing post-mortem examination from 2008 to 2015 were retrospectively identified from medical records. Thirteen of the dogs were excluded because they did not have thyroid gland saved at the time of post-mortem examination. One additional dog was excluded for a history of treatment for hypothyroidism. From 2015–2017, thyroid tissue was prospectively collected from an additional 11 dogs with diagnosis of gallbladder mucocele formation that underwent post-mortem examination. In total 29 thyroid lobes from 19 dogs with histologic diagnosis of gallbladder mucocele formation were included in the study. Ages of the 19 dogs ranged from 4 to 17 years (median, 12 years). Breeds represented included 5 Shetland Sheepdogs, 2 each of American Cocker Spaniels, Chihuahuas, and Mixed Breed Dogs, and 1 each of the following; Border Collie, Border Terrier, German Shepherd Dog, Golden Retriever, Lhasa Apso, Miniature Schnauzer, Shih Tzu, and West Highland White Terrier. Three of the dogs underwent thyroid function testing antemortem, none of which met criteria for diagnosis of hypothyroidism. Fourteen control dogs of predisposed age and breed that had gross and histologically normal appearing gallbladders and from whom thyroid tissue was archived at the time of post-mortem examination were also identified. Two dogs were excluded due to history of treatment for hypothyroidism. In total 20 thyroid lobes from 12 dogs with histologically normal appearing gallbladders were included in the study. Ages of the dogs ranged from 5 to 16 years (median, 12 years). Breeds represented included 5 American Cocker Spaniels, 4 Shetland Sheepdogs, and 3 Chihuahuas. None of the dogs had a history of thyroid function testing or thyroid hormone treatment. Gallbladder and thyroid tissue were additionally obtained from 4 apparently healthy research dogs and 5 shelter dogs immediately following euthanasia that was performed for reasons unrelated to the study. In total 15 thyroid lobes from 9 of these control dogs were included in the study. Research dogs were 4 year old mixed breed Foxhounds. Shelter dogs ranged in apparent age from young adult to geriatric and were represented by 3 American Staffordshire Terriers, a Husky, and a German Shepherd Dog. No control dogs had evidence of gallbladder mucocele formation.

Thyroid lobes from dogs with gallbladder mucocele formation did not differ significantly in light microscopic appearance of follicles or colloid compared to either group of control dogs. No significant differences between the two control groups were identified (Table 4).

**Table 4:** Results of blinded, semi-quantitative scoring of light microscopic findings of thyroid gland tissue obtained at the time of post-mortem examination from dogs with gallbladder mucocele formation and two groups of control dogs without gallbladder mucocele formation.

Disease Group	Number of follicles			Amount of colloid			Fatty infiltration				Inflammatory infiltrate				Mineral	Lipofuscin			
	Normal	Increased	Decreased	Normal	Increased	Decreased	None	Mild	Mod	Severe	None	Mild	Mod	Severe		Any	Mild	Mod	Severe
Gallbladder mucocele (n = 19)	12 (63%)	—	7 (37%)	7 (37%)	2 (10%)	10 (53%)	17 (89%)	2 (11%)	—	—	19 (100%)	—	—	—	11 (58%)*	8 (42%)*	2 (10%)	1 (5%)	5(26%)
No gallbladder mucocele																			
Older-aged, predisposedbreed (n = 12)	7 (58%)	—	5 (42%)	5 (42%)	—	7 (58%)	10 (83%)	2 (17%)	—	—	12 (100%)	—	—	—	2 (17%)	4 (33%)	1 (8%)	2 (17%)	1 (8%)
Apparently healthy purpose-bred and random-source (n = 9)	3 (33%)	—	6 (67%)	1 (11%)	—	8 (89%)	7 (78%)	1 (11%)	1 (11%)	—	8 (89%)	—	—	1 (11%)	0 (0%)	0 (0%)	—	—	—

Chi-squared test result compared to dogs with gallbladder mucocele formation

*p<0.05

**p<0.01.

A single control dog had evidence of severe lymphocytic thyroiditis, however inflammatory infiltrates were not observed in any glands from dogs with gallbladder mucocele formation. Overall, few dogs had evidence of fat within the thyroid gland and when present was mild with the exception of a moderate infiltrate in one control dog. Significantly more dogs with gallbladder mucocele formation had mineralization and lipofuscin present in thyroid tissue compared to glands from control dogs. An adenoma and carcinoma were identified in thyroid tissue from 2 dogs with gallbladder mucocele formation, respectively. An adenoma was identified in thyroid tissue from a single control dog.

**Fig 7:**
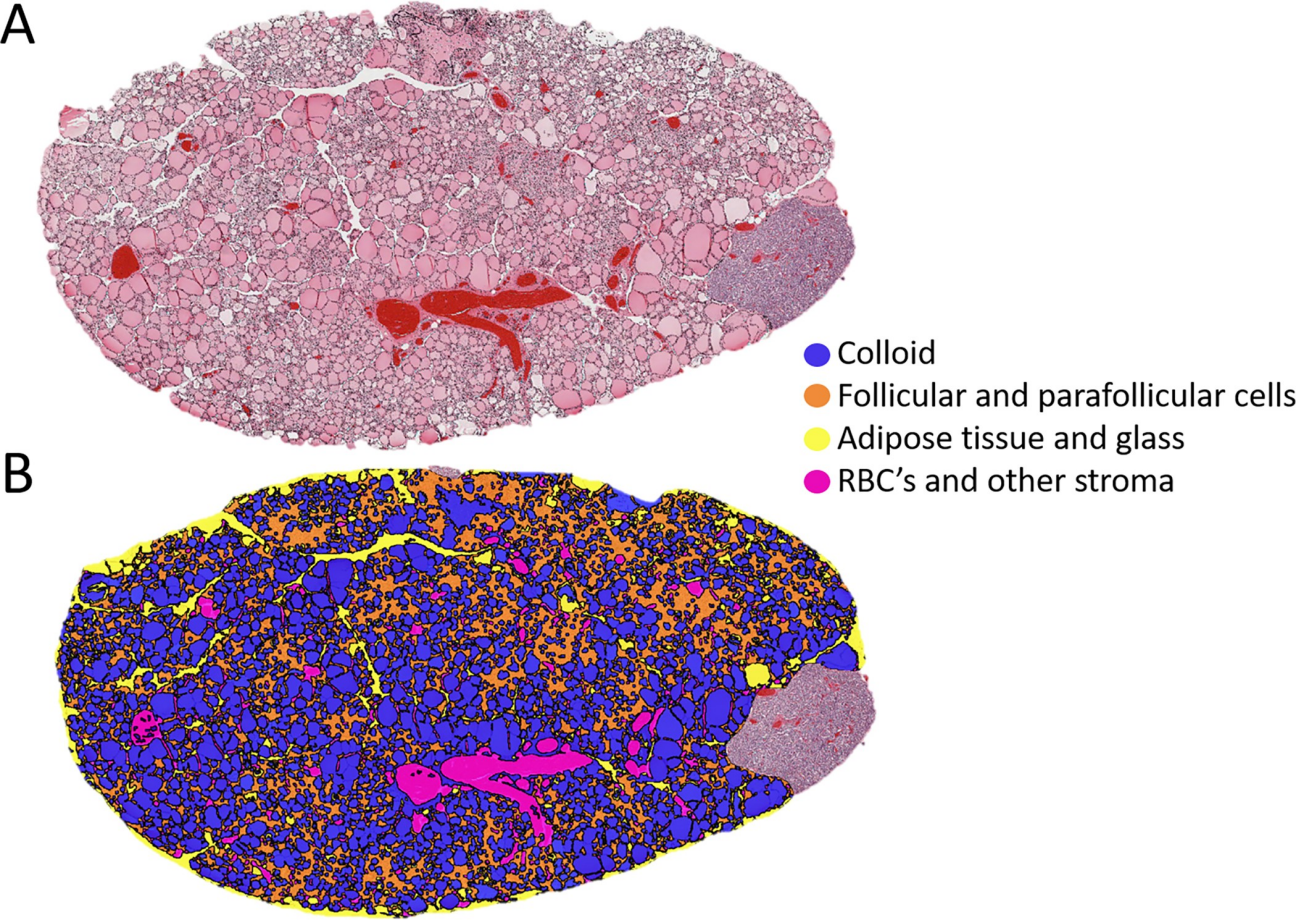
Histology (A) and computerized histomorphometry (B) of thyroid gland tissue from a 13 year old castrated male American Cocker Spaniel with gallbladder mucocele formation. Panel A represents the scanned in image of the original hematoxylin and eosin stained gland. Panel B demonstrates the computer-generated assignment of each tissue architectural feature into one of 4 different categories as shown.

Results of thyroid tissue analysis based on computer recognition of specified regions of interest (Fig 7) within the glands of dogs in each group are summarized in S5 Table. No significant differences between the 3 populations of dogs were observed.

## Discussion

### Adrenal function in dogs with gallbladder mucocele formation

Retrospective studies have identified a significant association between gallbladder mucocele formation and a diagnosis of hyperadrenocorticism in dogs [5, 13, 17]. The reported prevalence for hyperadrenocorticism diagnosis prior to or within 6 months following diagnosis of gallbladder mucocele formation in dogs is 23% and is based on results of either ACTH stimulation or low-dose dexamethasone suppression testing and supportive clinical signs[13]. In this study, we performed adrenal function testing on dogs that were diagnosed with gallbladder mucocele formation but had no obvious clinical signs or physical examination findings suggestive of hyperadrenocortism. Dogs with gallbladder mucocele formation had a significantly higher magnitude of increase in serum cortisol after stimulation with cosyntropin. This supports an increased capacity of the adrenal cortex for glucocorticoid synthesis although the increase was fairly modest. We observed no significant relationship between illness severity at the time of the study and magnitude of increase in serum cortisol, however an influence of chronic stress[57] in dogs with mucocele formation could not be reliably assessed and cannot be discounted. While progestogens appear to be able to promote gallbladder pathology similar to gallbladder mucocele formation in experimental studies[20–24], there was no evidence for a primary increase in endogenous progesterone synthesis by dogs with gallbladder mucocele formation in this study. However, this does not rule out the possibility of exposure to exogenous progestogens.

With the exception of DHEAS, no individual steroids were identified as increased above reference range significantly more often in dogs with gallbladder mucocele formation compared to control dogs. Only 3/30 (10%) dogs with gallbladder mucocele formation had a post-cosyntropin cortisol concentration exceeding the reference range calculated for use in this study (≥ 418 nmol/L) and also consistent with laboratory-based criteria for borderline (552–607 nmol/L [200–220 ng/ml]) or customary diagnosis of hyperadrenocorticism (i.e. ≥ 607 nmol/L [≥ 220 ng/ml])[58]. This is similar to the prevalence of hyperadrenocorticism reported as preexisting in dogs at the time of diagnosis of gallbladder mucocele formation (prevalence ranging from 8.9 to 14%)[6, 13, 17]. The comparable prevalence of post-cosyntropin hypercortisolemia in dogs at the time of diagnosis of gallbladder mucocele formation in this study does not support any major occult disturbance of glucocorticoid synthesis nor provide any justification for routine testing for hyperadrenocorticism in dogs with gallbladder mucocele formation that do not also have clinical signs of hypercortisolemia. In dogs with gallbladder mucocele formation, there was not a significant correlation between post-cosyntropin cortisol concentration and serum ALP activity. Instead, ALP was significantly correlated with serum biochemistry indicators of cholestasis such as total bilirubin, GGT, and cholesterol. The implication of this is that serum ALP activity in dogs with gallbladder mucocele formation may not be useful in assessing the possibility of underlying hyperadrenocorticism.

Perhaps the most interesting finding regarding adrenal function in dogs with gallbladder mucocele formation was that a significantly greater number of dogs had measureable and measurably higher concentrations of dehydroepiandrosterone sulfate (DHEAS) compared to control dogs. Few studies have included measurement of DHEAS in assessment of the steroidogenic response of the adrenal gland to cosyntropin administration in dogs[58, 59]. Those studies demonstrated that a measureable quantity of DHEAS is produced by the adrenal gland in clinically normal dogs and that serum concentrations of DHEAS are minimally responsive to cosyntropin stimulation. In this study, only a subset of control dogs (20%) and dogs with gallbladder mucocele formation (40%) had measureable serum DHEAS concentrations before or after stimulation with cosyntropin, likely reflecting the higher limit of detection of the mass spectrometry-based assay used in this study (39 nmol/L) compared to the radioimmunoassay used in prior reports (1.4 nmol/L[35]). Nonetheless, dogs with gallbladder mucocele formation had significantly higher concentrations of DHEAS than control dogs in this study and also when compared to previous reports of DHEAS concentrations in normal dogs[35] and dogs with hypercortisolemia[58]. The reason for increased serum DHEAS concentrations in dogs with gallbladder mucocele formation is unclear. The adrenal cortex normally produces three steroids with androgenic activity, dehydroepiandrosterone (DHEA), androstenedione, and testosterone. Concentrations of DHEA were below the limit of detection for all dogs with gallbladder mucocele formation and control dogs in this study. Inactivation of DHEA is mediated by sulfonation to form DHEAS, nearly all of which is synthesized in the adrenal cortex and then released into the circulation. As such, serum DHEAS concentration is considered to be a stable biomarker of adrenal androgen steroidogenesis[60]. High DHEAS and exaggerated adrenal steroidogenesis in the absence of an overt hypothalamic-pituitary-adrenal axis dysfunction is described in women with polycystic ovary syndrome but has an unclear pathogenesis[60]. It should also be considered that dogs with gallbladder mucocele formation could have an impaired excretion of DHEAS. DHEAS is transported by organic anion-transporting polypeptide (OATP) family members[61], ABCG2[62], and likely other efflux transporters[63] that play critical roles in excretion of endogenous compounds and xenobiotics. In particular, ABCG2 (aka Breast Cancer Resistance Protein, BCRP) resides in the hepatocyte canalicular membrane[62] and many ABCG2 substrates, including DHEAS, are products of phase II metabolism by co-expressed sulfotransferases. In the absence of ABCG2 activity these compounds can be increased in plasma and reduced in bile[63]. It is of interest that a number of compounds excreted by ACBG2, such as phytoestrogens[64] and riboflavin[65], were identified in a previous study to be significantly lower in the bile of dogs with gallbladder mucocele formation compared to control dogs[66]. Determining whether or not increases in DHEAS in dogs with gallbladder mucocele formation represent a biomarker of altered xenobiotic metabolism or transport versus an increase in androgen synthesis by the adrenal glands will require additional studies.

### Thyroid function, iodine homeostasis and thyroid gland pathology in dogs with gallbladder mucocele formation

The prevalence of hypothyroidism in dogs with gallbladder mucocele formation in prior studies is reported to range from 13 to 17%[5, 13, 17]. In this prospective study, we documented that 26% of dogs had thyroid profile test results arguably consistent with laboratory-based diagnostic criteria for hypothyroidism at the time of diagnosis of gallbladder mucocele formation. These dogs had no obvious clinical signs or physical examination findings suggestive of hypothyroidism and their test results were defined by having a low serum total T4 and an elevated serum TSH or a low serum FT_4_ concentration[13, 48, 49]. In view of the significant association between hypothyroidism and gallbladder mucocele formation reported in other studies[13, 17] and the observed prevalence of abnormal thyroid hormone test results in this study, it is worth considering that thyroid dysfunction in dogs with gallbladder mucocele formation may not simply be a coincidental disease process.

Several observations suggest that dogs with gallbladder mucocele formation and abnormal thyroid hormone test results in this study were unlikely to have primary hypothyroidism. First, no dogs in this study had positive test results for anti-thyroglobulin antibodies (TgAA). We would expect a prevalence of 50% positive for TgAA in this population if abnormal thyroid test results were due to subclinical immune-mediated thyroiditis[42, 43, 67]. Second, the lack of pathological findings such as inflammatory cell infiltrates, destruction of follicles, increase in adipose tissue, gland atrophy, or fibrosis[68, 69] in 29 thyroid lobes from 19 dogs with gallbladder mucocele formation in this study was noteworthy. While the sample size might be considered small, it should be sufficient to identify at least some stage in progression of thyroid disease if representing a population of dogs with a reported prevalence for diagnosis of primary hypothyroidism of 13–17%. This is particularly true if one considers the common assertion that more than 60 to 70% of thyroid tissue must be destroyed before changes are observed in laboratory measures of thyroid function[70]. In addition to histopathological examination, we performed an unbiased morphometric analysis of each thyroid tissue specimen and compared these findings to control dogs of predisposed age and breed as well as healthy dogs. Our rationale was that abnormal thyroid function in dogs with gallbladder mucocele formation would be reflected in the known structure-function relationship between activity of the thyroid gland and appearance of the follicular epithelium and colloid[71, 72]. Active thyroid tissue has small follicles with reduced colloid content lined by tall (active) follicular epithelial cells. Inactive thyroid tissue has large colloid-filled follicles lined by cuboidal (inactive) epithelium. In this study, we did not identify any significant or systematic differences between dogs with gallbladder mucocele formation and the other control groups. This finding indirectly suggests that the dogs with gallbladder mucocele formation, and whose thyroid glands were included in this analysis, had a similar potential for synthesizing and secreting thyroid hormones as compared to dogs without gallbladder mucocele formation. Therefore, it would seem unlikely that primary hypothyroidism or autoimmune thyroiditis was a major cause for abnormal thyroid hormone homeostasis in these dogs or somehow contributed to their gallbladder mucocele formation.

It is reasonable to suspect that abnormal thyroid hormone test results observed in dogs with gallbladder mucocele formation in this study could be attributed to concurrent non-thyroidal illness (NTI). Most dogs diagnosed with gallbladder mucocele formation have an illness-related indication for having undergone an abdominal ultrasound examination. We screened close to 100 dogs for inclusion in this study, over half of which were eliminated to minimize the impact of NTI or concurrent drug or thyroxine administration on thyroid hormone test results. Despite this, distinguishing between abnormal thyroid hormone homeostasis and the effect of NTI in these dogs is a formidable challenge. A number of studies have demonstrated that significant percentages of dogs with moderate to severe illness, and no clinical signs or laboratory findings suggestive of hypothyroidism, will have serum total T4, total T3, and/or FT_4_ in the hypothyroid range[44, 48, 71, 73–76]. Serum TSH appears to be least affected by NTI[44, 71]. To address an effect of NTI on thyroid hormone test results in this study, we stratified dogs by illness severity as previously described for dogs with NTI[44].

Consistent with prior studies of NTI in dogs, this study also demonstrated a significant association between illness severity and decreasing serum concentrations of total T4. However, a larger percentage of dogs with gallbladder mucocele formation had an abnormal total T4 (51% in this study versus 31[44] and 35%[74] in others) and this was observed at all degrees of illness severity (mild, moderate, severe; 37, 67, and 89% respectively) compared to reports of euthyroid dogs with NTI that were similarly stratified (8, 28, 60% respectively[44, 71, 77]). Prior studies reporting the effect of NTI on total T3 concentrations in dogs demonstrate a large variation in percentage of abnormal total T3 results ranging from 8% to 76%[44, 48, 74, 77], being largely related to the severity of illness in the dogs studied. However, in dogs with gallbladder mucocele formation, a much larger percentage of dogs had an abnormal total T3 at all degrees of illness severity (mild, moderate, severe; 86, 62, and 78% respectively) compared to reports of euthyroid dogs with NTI that were similarly stratified (3, 18, 27%, respectively)[44]. Moreover, the median total T3 in dogs with gallbladder mucocele formation was at or below the reference range in all categories of illness severity, while in other studies this was only observed in the most critically ill dogs[74, 75] or not at all[44]. In this study, 15% of dogs had serum total T4, total T3 or both below the limit of detection of the assay (i.e. 0 nmol/L). It is unlikely that the high prevalence of severely reduced total T3 concentrations in dogs with gallbladder mucocele formation in this study is due to primary hypothyroidism, because total T3 concentrations are not predictably reduced in hypothyroid dogs[48, 76]. Most dogs with gallbladder mucocele formation in this study had a concurrently low total T3 and total T4 (48.7%) which differs from a study of dogs with severe illness in which low total T3 alone was the most common finding (42.1%)[74]. Finally, 20% of dogs with gallbladder mucocele formation had a FT_3_ below reference range. Few reports exist on interpretation of FT_3_ values in dogs, however one study demonstrated no significant effect of systemic inflammatory response syndrome on FT_3_ measurements[75]. In contrast, prior studies report a significant association between illness severity and percentage of dogs with low FT_4_ (mild, moderate, severe; 7.6, 16.8, and 43.5% respectively)[44]. However, a low FT4 was much less common in dogs with gallbladder mucocele formation when similarly stratified (3, 6, 6% respectively). While an effect of NTI on thyroid hormone homeostasis in dogs with gallbladder mucocele formation undoubtedly contributed to some of the findings in this study, the severity of reduction in thyroid hormone concentrations, large percentage of dogs with abnormal values, and apparent lack of association with severity of NTI suggest that additional factors may be contributing to low serum thyroid hormone concentrations in these dogs. In considering other potential mechanisms for thyroid hormone disruption[78–80], this study found no correlation between decreasing serum concentrations of thyroid hormones and serum biochemistry values related to cholestasis (cholesterol, ALP, GGT and total bilirubin), post-cosyntropin cortisol or serum albumin concentration.

We have previously speculated that concurrent thyroid disruption, rather than coincidental primary hypothyroidism could be responsible for abnormal thyroid function in dogs with gallbladder mucocele formation[17]. Given the observation that experimental models of cystic fibrosis in piglets and ferrets have gallbladder pathology similar to gallbladder mucocele formation in dogs[25, 26] and a reported association between cystic fibrosis and iodine-deficiency hypothyroidism in people[28], we explored iodine homeostasis in dogs with gallbladder mucocele formation. Iodine is required for synthesis of thyroid hormones. After absorption by the gastrointestinal tract, iodide is transported into the colloid by thyroid follicular cells using the sodium-iodide symporter (NIS)[81] and chloride/iodide exchanger pendrin[82]. These transporters additionally depend on exchange of iodide with other ions such as Cl^-^, Na^+^, and K^+^[30, 82, 83]. Interruption of these transport mechanisms by thyroid disrupting compounds[84] or hereditary disease (such as Pendred syndrome[85] or cystic fibrosis[27, 28] can be associated with abnormal iodide uptake and impaired thyroid hormone synthesis[31]. Although concurrent gallbladder disease is not a typical feature of these conditions in people, gallbladder epithelial cells utilize many of the same transport mechanisms to promote biliary secretion[86–88].

To investigate the possibility of abnormal iodine homeostasis as an underlying cause for thyroid dysfunction in dogs with gallbladder mucocele formation, we measured urine iodine concentration and iodine-creatinine ratios (UICR) in the dogs in this study. In humans, urine iodine concentration is measured to gauge sufficiency of dietary iodine consumption as this value generally reflects iodine provided to the body in excess of thyroid gland requirements. In humans, urine iodine concentration < 100 μg/L is considered indicative of dietary deficiency and > 300 μg/L considered indicative of dietary excess. Dogs in this study had an average urine inorganic iodine concentration of 1,384 μg/L with a range from 27 to 5,473 μg/L. Therefore, rather than evidence of iodine deficiency, what we observed in this study was a higher than expected concentration of iodine in the urine of both control and gallbladder mucocele dogs. However, dogs with gallbladder mucocele formation had significantly higher urine iodine:creatinine ratios (UICR) compared to control dogs. We had speculated that high UICR in dogs with gallbladder mucocele formation could reflect disruption of iodide uptake by the thyroid gland, however the estimated quantity of iodine being excreted is considerably higher than could be achieved simply by interrupting thyroidal uptake of iodine, which is estimated to be 7.2 μg/kg/day in the adult beagle[89]. Finally, the finding of a non-significantly higher content of organic iodine in thyroid glands opportunistically collected from euthanized control dogs and dogs with gallbladder mucocele formation does not support the presence of thyroidal iodine deficiency.

The significance of high urine iodine concentrations in dogs in this study in general and the significantly higher UICRs in dogs with gallbladder mucocele formation remains unclear but is likely a reflection of dietary iodine intake. The reason why this might be relevant is that increasing dietary intake of iodine can be associated with development of subclinical hypothyroidism in people, especially among children, the elderly, and those with past or present thyroid disease or goitrogen exposure[90–97]. We are aware of only one study examining the effect of high dietary iodine on thyroid function in dogs. In the study, beagles that were fed a commercial diet containing 5.6 μg/g dry weight of iodine (estimated to provide 1,200 to 1,800 μg/day as fed) developed significantly lower total T4, FT_4_, and higher TSH concentrations compared to dogs fed a diet containing 0.25 μg/g of iodine (100 μg/day as fed)[98]. Based on the quantity of iodine measured in the urine of dogs in this study, many were likely consuming diets providing equivalent or more iodine than was associated with subclinical hypothyroidism in the beagle study. Older studies report iodine content ranging from 0.5 to 5.6 μg/g dry weight with an estimated daily intake corresponding to 400 to 3,750 μg/day as fed to the dogs studied[89, 98]. A more recent study reports inorganic iodine content ranging from 0.86 to 4.05 μg/g dry weight, however the additional presence of organic iodine in the form of intact thyroid hormones (T4 and T3) and thyroid hormone precursors (MIT and DIT) raised the total iodine content to 2.1 to 136 μg/g dry weight[99]. Many of the diets and treats examined in this latter study were consumed by dogs diagnosed with dietary thyrotoxicosis[100], presumably due to the presence of intact thyroid hormones in the diet. Importantly, as regards the present study, it is not clear if there is any direct link between the high UICR, the observed abnormal thyroid hormone concentrations, and pathogenesis of gallbladder mucocele formation in the dogs reported here. Indeed, we observed no direct correlation between UICR and thyroid hormone concentrations in dogs with gallbladder mucocele formation.

### Study limitations

There are several important limitations to this study. The first is that the HPLC-MS/MS assay used for measurement of steroid hormones in this study has not been specifically validated for use in dogs. Within our objective of discovering any differences in steroid hormone metabolism between control dogs and dogs with gallbladder mucocele formation, we selected this particular assay based on the ability to simultaneously and quantitatively measure the largest number of different steroid hormones in a single commercial assay. While expected ranges in concentration for commonly measured steroids were generally obtained, for some steroids the assay lacked sensitivity (e.g. testosterone, aldosterone), generated values lower than prior publications (e.g. androstendione[35, 36]), or failed to detect an expected steroid (estradiol[101]). Accordingly, the reported assay results should be considered as a means for comparing the two populations of dogs included in this study and not as having validated diagnostic value.

An additional limitation to this study is that we did not also perform adrenal or thyroid function testing on a population of sick dogs without gallbladder mucocele formation. In our opinion, this decision is justified by numerous prior studies having clearly established the expected impact of concurrent illness on results of thyroid function testing in dogs[44, 48, 71, 73–76] and our use of an established scoring system[44] to directly examine the significance of illness severity on thyroid hormone test results in our population of dogs. Nonetheless, the current study design could not quantify the influence of chronic stress in these dogs, particular in regards to an influence on the results of steroid hormone testing. It would have been interesting to conduct follow up thyroid function testing in dogs that ultimately recovered from their clinical illness, however this was beyond the scope of our study. Ideally this study would have included a direct assessment of thyroid function such as TSH stimulation testing or measurement of thyroidal uptake of ^99m^TcO_4_-[73], however this was cost prohibitive and considered not to be in the best interest of these clinical patients. Ultrasonographic examination of the thyroid gland was not performed and ultrasound examination of the adrenal glands was inconsistently performed in the dogs in this study and would likely have added additional value to these studies. Ultrasonography would be worth considering as an adjunct to assessment of thyroid and adrenal function in dogs at the time of diagnosis of gallbladder mucocele formation[102–105]. Ultrasonographic examination of the gallbladder is not 100% specific for diagnosis of mucocele formation and therefore misclassification of some dogs in this study is possible. Finally, a major limitation of our studies conducted on archival thyroid tissue from dogs with gallbladder mucocele formation is that the majority of these dogs did not undergo thyroid hormone testing. This approach was unavoidable because thyroid gland biopsy was not considered a justifiable approach to procuring tissue from the live dogs participating in this study. A similar limitation applied to our measurement of thyroid iodine content which required collection of fresh thyroid tissue and therefore limited to dogs with gallbladder mucocele formation that were euthanized over the time course of the study. The majority of these dogs did not have concurrent thyroid hormone or urine iodine measurements performed. Control thyroid tissue for organic iodine measurement was largely, but not exclusively, obtained from younger-aged, healthy dogs.

### Application of findings

Based on the high prevalence of serum thyroid hormone abnormalities in this study and others[13] and indirect support for a therapeutic effect of levothyroxine in some dogs with gallbladder mucocele formation[34], diagnostic testing for hypothyroidism in dogs with gallbladder mucocele formation seems warranted. What remains unclear is whether or not these dogs actually have primary hypothyroidism versus NTI or some other form of thyroid disruption. This study did not provide strong immunological (TgAA) or histological evidence for a high prevalence of primary hypothyroidism in dogs with gallbladder mucocele formation and the contribution of NTI in this study remains unresolved. Given the confounding influence of NTI in dogs with gallbladder mucocele formation, testing should be performed, if possible, prior to onset or after resolution of clinical illness, be based on results of a comprehensive thyroid profile, and ideally undertaken in dogs with other supportive clinical signs. This study did not find any correlation between high serum cholesterol and decreases in serum concentration of thyroid hormones in dogs with gallbladder mucocele formation. These findings suggest that dogs of an age and breed at risk for gallbladder mucocele formation and recognized to have high serum cholesterol should undergo abdominal ultrasonography to rule out gallbladder mucocele formation in addition to comprehensive thyroid hormone testing. The present finding that dogs with gallbladder mucocele formation have higher UICR compared to control dogs is of undetermined significance but worthy of additional investigation.

## Supporting information

S1 Table Number (%) of dogs, before administration of cosyntropin, that had steroid concentrations above the upper limit of the 95% reference interval established using control dog steroid concentrations. The 90% confidence interval (CI) for the upper limit value of each individual steroid is also shown.
(DOCX)

S2 Table Number (%) of dogs, after administration of cosyntropin, that had steroid concentrations above the upper limit of the 95% reference interval established using control dog steroid concentrations. The 90% confidence interval (CI) for the upper limit value of each individual steroid is also shown.
(DOCX)

S3 Table Correlation matrix comparing serum biochemistry analysis, thyroid hormone, post-cosyntropin cortisol, endogenous TSH and ACTH, and UICR test results in control dogs. (DOCX)

S4 Table Correlation matrix comparing serum biochemistry analysis, thyroid hormone, post-cosyntropin cortisol, endogenous TSH and ACTH, and UICR test results in dogs with gallbladder mucocele formation. (DOCX)

S5 Table (DOCX)

S1 Fig Measurements of serum cortisol concentration before and 1 hour after administration of cosyntropin to control dogs and dogs diagnosed with gallbladder mucocele formation. (DOCX)
