# Supplementary material for: Investigation of adrenal and thyroid gland dysfunction in dogs with ultrasonographic diagnosis of gallbladder mucocele formation
Source: PLoS One. 2019 Feb 27;14(2):e0212638. doi: 10.1371/journal.pone.0212638 (PMC6392329; doi:10.1371/journal.pone.0212638)
Supplement: S1 Table — The 90% confidence interval (CI) for the upper limit value of each individual steroid is also shown. (DOCX) [file pone.0212638.s001.docx]

**Supporting information**

**S1 Table.** Number (%) of dogs, before administration of cosyntropin, that had steroid concentrations above the upper limit of the 95% reference interval established using control dog steroid concentrations. The 90% confidence interval (CI) for the upper limit value of each individual steroid is also shown.

| **Steroid (nmol/L)** | **Reference Interval Upper Limit** | **90% Bootstrap CI** | **Number (%) of dogs with values above Reference Interval** | |
| --- | --- | --- | --- | --- |
|  |  |  | **Control** | **Mucocele** |
| 11-Deoxycorticosterone | 3.6 | 2.6-4.7 | 1 (3) | 0 (0) |
| 11-Deoxycortisol | 37.0 | 24.0-46.6 | 3 (10) | 0 (0) |
| Corticosterone | 33.7 | 24.2-42.1 | 2 (7) | 0 (0) |
| Cortisol | 215.0 | 169.0-261.1 | 1 (3) | 0 (0) |
| Cortisone | 33.2 | 28.2-38.1 | 3 (10) | 0 (0) |
| Progesterone | 1.08 | 0.72-1.36 | 1 (3) | 0 (0) |
| 17α-Hydroxyprogesterone | 1.0 | 0.74-1.3 | 0 (0) | 0 (0) |
| Androstenedione | 0.08 | 0.023-0.11 | NA | NA |
| Any steroid above reference interval | | | 4 (13) | 0 (0) |
| Median (range) number of different steroids above reference interval | | | 3 (1-4) | 0 |

NA, not analyzed due to >60% of dogs with values < limit of detection
