# Supplementary material for: Investigation of adrenal and thyroid gland dysfunction in dogs with ultrasonographic diagnosis of gallbladder mucocele formation
Source: PLoS One. 2019 Feb 27;14(2):e0212638. doi: 10.1371/journal.pone.0212638 (PMC6392329; doi:10.1371/journal.pone.0212638)
Supplement: S2 Table — The 90% confidence interval (CI) for the upper limit value of each individual steroid is also shown. (DOCX) [file pone.0212638.s002.docx]

**Supporting information**

**S2 Table.** Number (%) of dogs, after administration of cosyntropin, that had steroid concentrations above the upper limit of the 95% reference interval established using control dog steroid concentrations. The 90% confidence interval (CI) for the upper limit value of each individual steroid is also shown.

| **Steroid (nmol/L)** | **Reference Interval Upper Limit** | **90% Bootstrap CI** | **Number (%) of dogs with values above Reference Interval** | |
| --- | --- | --- | --- | --- |
|  |  |  | **Control** | **Mucocele** |
| 11-Deoxycorticosterone | 17.0 | 13.4-19.9 | 1 (3) | 3 (10) |
| 11-Deoxycortisol | 172 | 127-221 | 2 (7) | 1 (3) |
| Corticosterone | 88.0 | 76.5-97.4 | 1 (3) | 3 (10) |
| Cortisol | 418 | 378-454 | 0 (0) | 3 (10) |
| Cortisone | 47.8 | 43.8-51.7 | 1 (3) | 3 (10) |
| Progesterone | 6.4 | 5.1-7.5 | 0 (0) | 2 (7) |
| 17α-Hydroxyprogesterone | 8.0 | 5.8-9.6 | 1 (3) | 3 (10) |
| Androstenedione | 0.48 | 0.30-0.65 | 1 (3) | 2 (7) |
| Any steroid above reference interval | | | 5 (17) | 7 (23) |
| Median (range) number of different steroids above reference interval | | | 1 (1-3) | 3 (1-4) |
