# Supplementary material for: Investigation of adrenal and thyroid gland dysfunction in dogs with ultrasonographic diagnosis of gallbladder mucocele formation
Source: PLoS One. 2019 Feb 27;14(2):e0212638. doi: 10.1371/journal.pone.0212638 (PMC6392329; doi:10.1371/journal.pone.0212638)
Supplement: S3 Table — (DOCX) [file pone.0212638.s003.docx]

**Supporting information**

**S3 Table.** Correlation matrix comparing serum biochemistry analysis, thyroid hormone, post-cosyntropin cortisol, endogenous TSH and ACTH, and UICR test results in control dogs. For each pair of variables, cell contents reflect (from top to bottom) the Pearson’s Product Moment Correlation Coefficient, p-value, and number of dogs. Shaded cells represent correlations remaining statistically significant at a Benjamini-Hochberg false discovery rate of <0.15.

|  | **Cholesterol** | **ALP** | **Bilirubin** | **Albumin** | **BUN** | **Creatinine** | **Lipase** | **Amylase** | **TT3** | **TT4** | **FT3** | **FT4** | **TSH** | **UICR** | **Cortisol** | **ACTH** |
| --- | --- | --- | --- | --- | --- | --- | --- | --- | --- | --- | --- | --- | --- | --- | --- | --- |
| **Age** | -0.049 | 0.141 | -0.356 | -0.333 | **0.550** | 0.230 | 0.0735 | **0.543** | -0.331 | -0.181 | -0.368 | -0.206 | 0.306 | -0.214 | -0.150 | 0.206 |
|  | 0.800 | 0.467 | 0.058 | 0.077 | **0.002** | 0.230 | 0.705 | **0.0023** | 0.079 | 0.346 | 0.076 | 0.285 | 0.107 | 0.274 | 0.456 | 0.302 |
|  | 29 | 29 | 29 | 29 | **29** | 29 | 29 | **29** | 29 | 29 | 24 | 29 | 29 | 28 | 27 | 27 |
| **Cholesterol** |  | 0.081 | 0.052 | 0.224 | 0.304 | 0.226 | 0.386 | 0.0051 | 0.387 | 0.072 | 0.492 | 0.294 | 0.339 | 0.073 | 0.119 | -0.085 |
|  |  | 0.672 | 0.783 | 0.233 | 0.103 | 0.230 | 0.035 | 0.979 | 0.035 | 0.706 | 0.013 | 0.115 | 0.066 | 0.708 | 0.556 | 0.666 |
|  |  | 30 | 30 | 30 | 30 | 30 | 30 | 30 | 30 | 30 | 25 | 30 | 30 | 29 | 28 | 28 |
| **ALP** |  |  | -0.106 | 0.440 | 0.123 | -0.245 | 0.164 | 0.124 | 0.376 | 0.171 | 0.196 | 0.303 | 0.0915 | -0.024 | **0.484** | 0.189 |
|  |  |  | 0.576 | 0.015 | 0.517 | 0.192 | 0.386 | 0.515 | 0.04 | 0.366 | 0.348 | 0.104 | 0.630 | 0.901 | **0.0091** | 0.336 |
|  |  |  | 30 | 30 | 30 | 30 | 30 | 30 | 30 | 30 | 25 | 30 | 30 | 29 | **28** | 28 |
| **Bilirubin** |  |  |  | 0.127 | 0.026 | -0.092 | 0.202 | -0.301 | 0.304 | 0.113 | 0.303 | 0.305 | -0.127 | -0.060 | 0.147 | -0.238 |
|  |  |  |  | 0.504 | 0.892 | 0.627 | 0.284 | 0.106 | 0.102 | 0.553 | 0.141 | 0.101 | 0.504 | 0.757 | 0.455 | 0.223 |
|  |  |  |  | 30 | 30 | 30 | 30 | 30 | 30 | 30 | 25 | 30 | 30 | 29 | 28 | 28 |
| **Albumin** |  |  |  |  | -0.241 | -0.238 | -0.070 | -0.307 | **0.575** | **0.466** | 0.359 | 0.324 | 0.0031 | 0.220 | 0.135 | -0.051 |
|  |  |  |  |  | 0.199 | 0.206 | 0.715 | 0.099 | **0.00088** | **0.0095** | 0.078 | 0.081 | 0.987 | 0.250 | 0.495 | 0.796 |
|  |  |  |  |  | 30 | 30 | 30 | 30 | **30** | **30** | 25 | 30 | 30 | 29 | 28 | 28 |
| **BUN** |  |  |  |  |  | 0.383 | 0.192 |  | -0.190 | -0.208 | 0.153 | 0.084 | 0.136 | -0.176 | 0.276 | 0.101 |
|  |  |  |  |  |  | 0.036 | 0.311 |  | 0.314 | 0.269 | 0.466 | 0.660 | 0.472 | 0.361 | 0.156 | 0.608 |
|  |  |  |  |  |  | 30 | 30 |  | 30 | 30 | 25 | 30 | 30 | 29 | 28 | 28 |
| **Creatinine** |  |  |  |  |  |  | 0.160 | 0.052 | -0.249 | -0.334 | 0.036 | -0.102 | 0.305 | -0.173 | -0.062 | -0.039 |
|  |  |  |  |  |  |  | 0.397 | 0.786 | 0.185 | 0.072 | 0.864 | 0.590 | 0.102 | 0.368 | 0.753 | 0.843 |
|  |  |  |  |  |  |  | 30 | 30 | 30 | 30 | 25 | 30 | 30 | 29 | 28 | 28 |
| **Lipase** |  |  |  |  |  |  |  | 0.211 | 0.297 | -0.033 | 0.376 | 0.172 | 0.318 | -0.111 | 0.107 | 0.160 |
|  |  |  |  |  |  |  |  | 0.264 | 0.111 | 0.861 | 0.064 | 0.363 | 0.087 | 0.568 | 0.589 | 0.416 |
|  |  |  |  |  |  |  |  | 30 | 30 | 30 | 25 | 30 | 30 | 29 | 28 | 28 |
| **Amylase** |  |  |  |  |  |  |  |  | -0.336 | -0.121 | -0.125 | -0.263 | 0.232 | 0.107 | -0.077 | 0.288 |
|  |  |  |  |  |  |  |  |  | 0.069 | 0.524 | 0.552 | 0.161 | 0.216 | 0.580 | 0.697 | 0.138 |
|  |  |  |  |  |  |  |  |  | 30 | 30 | 25 | 30 | 30 | 29 | 28 | 28 |
| **TT3** |  |  |  |  |  |  |  |  |  | **0.455** | 0.379 | 0.360 | -0.135 | 0.242 | 0.167 | -0.232 |
|  |  |  |  |  |  |  |  |  |  | **0.011** | 0.062 | 0.051 | 0.477 | 0.205 | 0.395 | 0.236 |
|  |  |  |  |  |  |  |  |  |  | **30** | 25 | 30 | 30 | 29 | 28 | 28 |
| **TT4** |  |  |  |  |  |  |  |  |  |  | 0.432 | **0.542** | -0.430 | **0.528** | -0.181 | -0.186 |
|  |  |  |  |  |  |  |  |  |  |  | 0.031 | **0.002** | 0.018 | **0.0033** | 0.356 | 0.343 |
|  |  |  |  |  |  |  |  |  |  |  | 25 | **30** | 30 | **29** | 28 | 28 |
| **FT3** |  |  |  |  |  |  |  |  |  |  |  | **0.755** | 0.152 | 0.207 | 0.458 | -0.036 |
|  |  |  |  |  |  |  |  |  |  |  |  | **1.3x10^-5^** | 0.470 | 0.320 | 0.028 | 0.871 |
|  |  |  |  |  |  |  |  |  |  |  |  | **25** | 25 | 25 | 23 | 23 |
| **FT4** |  |  |  |  |  |  |  |  |  |  |  |  | -0.035 | 0.150 | 0.366 | 0.103 |
|  |  |  |  |  |  |  |  |  |  |  |  |  | 0.853 | 0.438 | 0.055 | 0.604 |
|  |  |  |  |  |  |  |  |  |  |  |  |  | 30 | 29 | 28 | 28 |
| **TSH** |  |  |  |  |  |  |  |  |  |  |  |  |  | -0.241 | -0.065 | **0.497** |
|  |  |  |  |  |  |  |  |  |  |  |  |  |  | 0.208 | 0.742 | **0.0071** |
|  |  |  |  |  |  |  |  |  |  |  |  |  |  | 29 | 28 | **28** |
| **UICR** |  |  |  |  |  |  |  |  |  |  |  |  |  |  | -0.302 | -0.257 |
|  |  |  |  |  |  |  |  |  |  |  |  |  |  |  | 0.126 | 0.195 |
|  |  |  |  |  |  |  |  |  |  |  |  |  |  |  | 27 | 27 |
| **Cortisol** |  |  |  |  |  |  |  |  |  |  |  |  |  |  |  | 0.0302 |
|  |  |  |  |  |  |  |  |  |  |  |  |  |  |  |  | 0.879 |
|  |  |  |  |  |  |  |  |  |  |  |  |  |  |  |  | 28 |
