# Supplementary material for: Investigation of adrenal and thyroid gland dysfunction in dogs with ultrasonographic diagnosis of gallbladder mucocele formation
Source: PLoS One. 2019 Feb 27;14(2):e0212638. doi: 10.1371/journal.pone.0212638 (PMC6392329; doi:10.1371/journal.pone.0212638)
Supplement: S4 Table — (DOCX) [file pone.0212638.s004.docx]

**Supporting information**

**S4 Table.** Correlation matrix comparing serum biochemistry analysis, thyroid hormone, post-cosyntropin cortisol, endogenous TSH and ACTH, and UICR test results in dogs with gallbladder mucocele formation. For each pair of variables, cell contents reflect (from top to bottom) the Pearson’s Product Moment Correlation Coefficient, p-value, and number of dogs. Shaded cells represent correlations remaining statistically significant at a Benjamini-Hochberg false discovery rate of <0.15.

|  | **Cholesterol** | **ALP** | **GGT** | **Bilirubin** | **Albumin** | **BUN** | **Creatinine** | **Lipase** | **Amylase** | **TT3** | **TT4** | **FT3** | **FT4** | **TSH** | **UICR** | **Cortisol** | **ACTH** |
| --- | --- | --- | --- | --- | --- | --- | --- | --- | --- | --- | --- | --- | --- | --- | --- | --- | --- |
| **Age** | 0.0736 | -0.147 | -0.0063 | 0.0074 | -0.143 | **0.383** | 0.319 | -0.0065 | 0.031 | -0.166 | -0.261 | -0.065 | **-0.450** | **0.492** | **0.426** | -0.0117 | 0.177 |
|  | 0.661 | 0.378 | 0.970 | 0.965 | 0.392 | **0.018** | 0.051 | 0.969 | 0.854 | 0.312 | 0.109 | 0.732 | **0.0066** | **0.0027** | **0.015** | 0.951 | 0.350 |
|  | 38 | 38 | 38 | 38 | 38 | **38** | 38 | 38 | 38 | 39 | 39 | 30 | **35** | **35** | **32** | 30 | 30 |
| **Cholesterol** |  | **0.394** | 0.260 | **0.406** | **0.377** | -0.254 | -0.271 | -0.0140 | -0.0108 | **0.436** | 0.189 | **0.506** | 0.0525 | -0.075 | -0.022 | 0.0236 | -0.099 |
|  |  | **0.014** | 0.115 | **0.011** | **0.02** | 0.124 | 0.100 | 0.934 | 0.949 | **0.0062** | 0.256 | **0.0051** | 0.768 | 0.673 | 0.904 | 0.901 | 0.602 |
|  |  | **38** | 38 | **38** | **38** | 38 | 38 | 38 | 38 | **38** | 38 | **29** | 34 | 34 | 31 | 30 | 30 |
| **ALP** |  |  | **0.400** | **0.627** | -0.0633 | -0.192 | -0.257 | 0.0553 | -0.0191 | 0.0703 | -0.160 | 0.0506 | 0.049 | -0.114 | -0.190 | 0.084 | -0.190 |
|  |  |  | **0.013** | **2.5 X 10^-5^** | 0.706 | 0.247 | 0.119 | 0.742 | 0.909 | 0.675 | 0.337 | 0.794 | 0.783 | 0.519 | 0.306 | 0.657 | 0.315 |
|  |  |  | **38** | **38** | 38 | 38 | 38 | 38 | 38 | 38 | 38 | 29 | 34 | 34 | 31 | 30 | 30 |
| **GGT** |  |  |  | 0.263 | 0.148 | -0.189 | -0.197 | 0.0679 | 0.149 | 0.019 | -0.202 | -0.00435 | -0.104 | -0.0332 | 0.177 | **0.430** | -0.0373 |
|  |  |  |  | 0.111 | 0.376 | 0.256 | 0.235 | 0.685 | 0.373 | 0.910 | 0.223 | 0.982 | 0.559 | 0.852 | 0.340 | **0.018** | 0.845 |
|  |  |  |  | 38 | 38 | 38 | 38 | 38 | 38 | 38 | 38 | 29 | 34 | 34 | 31 | **30** | 30 |
| **Bilirubin** |  |  |  |  | -0.0429 | -0.143 | -0.201 | 0.0312 | -0.158 | 0.0664 | -0.142 | -0.0299 | 0.228 | -0.0431 | -0.340 | -0.032 | -0.170 |
|  |  |  |  |  | 0.798 | 0.390 | 0.226 | 0.853 | 0.343 | 0.692 | 0.396 | 0.878 | 0.196 | 0.809 | 0.0614 | 0.868 | 0.370 |
|  |  |  |  |  | 38 | 38 | 38 | 38 | 38 | 38 | 38 | 29 | 34 | 34 | 31 | 30 | 30 |
| **Albumin** |  |  |  |  |  | **-0.414** | **-0.426** | -0.337 | -0.318 | **0.542** | **0.525** | **0.596** | 0.170 | 0.047 | 0.0365 | 0.0095 | -0.0313 |
|  |  |  |  |  |  | **0.0097** | **0.0077** | 0.0385 | 0.0514 | **0.00044** | **0.00071** | **0.00065** | 0.335 | 0.791 | 0.845 | 0.960 | 0.870 |
|  |  |  |  |  |  | **38** | **38** | 38 | 38 | **38** | **38** | **29** | 34 | 34 | 31 | 30 | 30 |
| **BUN** |  |  |  |  |  |  | **0.926** | -0.121 | 0.0403 | -0.215 | -0.274 | -0.331 | -0.237 | 0.0598 | 0.110 | -0.0432 | 0.336 |
|  |  |  |  |  |  |  | **9 X 10^-17^** | 0.471 | 0.810 | 0.194 | 0.0963 | 0.0793 | 0.178 | 0.737 | 0.557 | 0.821 | 0.07 |
|  |  |  |  |  |  |  | **38** | 38 | 38 | 38 | 38 | 29 | 34 | 34 | 31 | 30 | 30 |
| **Creatinine** |  |  |  |  |  |  |  | -0.158 | 0.0891 | -0.319 | -0.294 | -0.354 | -0.352 | 0.0066 | 0.151 | 0.0914 | 0.332 |
|  |  |  |  |  |  |  |  | 0.344 | 0.595 | 0.051 | 0.073 | 0.0592 | 0.041 | 0.971 | 0.418 | 0.631 | 0.073 |
|  |  |  |  |  |  |  |  | 38 | 38 | 38 | 38 | 29 | 34 | 34 | 31 | 30 | 30 |
| **Lipase** |  |  |  |  |  |  |  |  | **0.672** | -0.0937 | -0.170 | -0.0990 | 0.0904 | -0.0630 | 0.00300 | 0.204 | 0.228 |
|  |  |  |  |  |  |  |  |  | **3.9 X 10^-6^** | 0.576 | 0.309 | 0.609 | 0.611 | 0.724 | 0.987 | 0.281 | 0.227 |
|  |  |  |  |  |  |  |  |  | **38** | 38 | 38 | 29 | 34 | 34 | 31 | 30 | 30 |
| **Amylase** |  |  |  |  |  |  |  |  |  | -0.105 | -0.177 | -0.0285 | -0.169 | -0.177 | **0.454** | **0.622** | 0.0901 |
|  |  |  |  |  |  |  |  |  |  | 0.529 | 0.288 | 0.883 | 0.340 | 0.317 | **0.010** | **0.00024** | 0.636 |
|  |  |  |  |  |  |  |  |  |  | 38 | 38 | 29 | 34 | 34 | **31** | **30** | 30 |
| **TT3** |  |  |  |  |  |  |  |  |  |  | **0.761** | **0.835** | **0.591** | -0.304 | -0.143 | -0.254 | -0.255 |
|  |  |  |  |  |  |  |  |  |  |  | **1.8 X 10^-8^** | **1 X 10^-7^** | **0.00019** | 0.076 | 0.435 | 0.175 | 0.173 |
|  |  |  |  |  |  |  |  |  |  |  | **39** | **30** | **35** | 35 | 32 | 30 | 30 |
| **TT4** |  |  |  |  |  |  |  |  |  |  |  | **0.736** | **0.463** | -0.173 | 0.0595 | -0.218 | -0.163 |
|  |  |  |  |  |  |  |  |  |  |  |  | **3.6 X 10^-6^** | **0.0051** | 0.319 | 0.746 | 0.247 | 0.390 |
|  |  |  |  |  |  |  |  |  |  |  |  | **30** | **35** | 35 | 32 | 30 | 30 |
| **FT3** |  |  |  |  |  |  |  |  |  |  |  |  | 0.392 | -0.138 | -0.0462 | -0.221 | -0.260 |
|  |  |  |  |  |  |  |  |  |  |  |  |  | 0.032 | 0.468 | 0.830 | 0.288 | 0.209 |
|  |  |  |  |  |  |  |  |  |  |  |  |  | 30 | 30 | 24 | 25 | 25 |
| **FT4** |  |  |  |  |  |  |  |  |  |  |  |  |  | **-0.442** | -0.392 | -0.306 | -0.0023 |
|  |  |  |  |  |  |  |  |  |  |  |  |  |  | **0.0079** | 0.039 | 0.100 | 0.990 |
|  |  |  |  |  |  |  |  |  |  |  |  |  |  | **35** | 28 | 30 | 30 |
| **TSH** |  |  |  |  |  |  |  |  |  |  |  |  |  |  | 0.308 | 0.191 | 0.302 |
|  |  |  |  |  |  |  |  |  |  |  |  |  |  |  | 0.110 | 0.311 | 0.105 |
|  |  |  |  |  |  |  |  |  |  |  |  |  |  |  | 28 | 30 | 30 |
| **UICR** |  |  |  |  |  |  |  |  |  |  |  |  |  |  |  | **0.654** | 0.0623 |
|  |  |  |  |  |  |  |  |  |  |  |  |  |  |  |  | **0.00053** | 0.772 |
|  |  |  |  |  |  |  |  |  |  |  |  |  |  |  |  | **24** | 24 |
| **Cortisol** |  |  |  |  |  |  |  |  |  |  |  |  |  |  |  |  | 0.216 |
|  |  |  |  |  |  |  |  |  |  |  |  |  |  |  |  |  | 0.251 |
|  |  |  |  |  |  |  |  |  |  |  |  |  |  |  |  |  | 30 |
