# Supplementary material for: Investigation of adrenal and thyroid gland dysfunction in dogs with ultrasonographic diagnosis of gallbladder mucocele formation
Source: PLoS One. 2019 Feb 27;14(2):e0212638. doi: 10.1371/journal.pone.0212638 (PMC6392329; doi:10.1371/journal.pone.0212638)
Supplement: S5 Table — (DOCX) [file pone.0212638.s005.docx]

**Supporting information**

**S5 Table** Results of quantitative histomorphometry performed on thyroid gland tissue from dogs with gallbladder mucocele formation and two groups of control dogs without gallbladder mucocele formation.

| **Histomorphometric variable** | **Gallbladder Mucocele**  **(29 lobes from 19 dogs)** | | **No Gallbladder Mucocele** | | | |
| --- | --- | --- | --- | --- | --- | --- |
|  |  |  | **Older-aged,**  **predisposed breeds**  **(20 lobes from 12 dogs)** | | **Apparently healthy purpose-bred and random-source**  **(15 lobes from 9 dogs)** | |
|  | **Median** | **25% to 75%** | **Median** | **25% to 75%** | **Median** | **25% to 75%** |
| Relative percent tissue area |  |  |  |  |  |  |
| Colloid % | 37.5 | 24.5-42.7 | 35.5 | 24.4-45.7 | 35.4 | 28.7-41.9 |
| Follicular and parafollicular cells % | 42.6 | 28.5-54.1 | 43.7 | 21.7-56.8 | 43.4 | 33.5-54.7 |
| Adipose tissue and glass % | 12.0 | 8.0-16.1 | 7.8 | 4.7-12.4 | 14.2 | 9.4-18 |
| All other stroma % | 5.7 | 1.8-1.3 | 5.1 | 2.9-12.3 | 5.3 | 2.1-7.7 |
| No. colloid follicles per tissue area (µm^2^) | 8.3 x 10^-5^ | 7-10 x 10^-5^ | 9.2 x 10^-5^ | 6-10 x 10^-5^ | 9.7 x 10^-5^ | 6-10 x 10^-5^ |
| No. colloid follicles per colloid area (µm^2^) | 2.5 x 10^-4^ | 1-4 x 10^-4^ | 2.7 x 10^-4^ | 2-4 x 10^-4^ | 2.8 x 10^-4^ | 2-4 x 10^-4^ |
| No. nuclei per tissue area (µm^2^) | 1.0 x 10^-5^ | 0.5-3 x 10^-5^ | 1.2 x 10^-5^ | 0.3-3 x 10^-5^ | 1.0 x 10^-5^ | 0.6-2 x 10^-5^ |
| Height of follicular epithelium (µm) | 7.175 | 6.336-8.326 | 7.640 | 6.974-9.664 | 7.893 | 7.477-8.044 |
| Area per follicle (µm^2^) | 3,917 | 2,437-5482 | 3,732 | 2,403-4,283 | 3,503 | 2,510-4,940 |
| Eosin intensity of colloid | 0.191 | 0.12-0.26 | 0.211 | 0.13-0.25 | 0.15 | 0.072-0.25 |
| Hematoxylin intensity of colloid | 0.043 | 0.034-0.059 | 0.036 | 0.033-0.045 | 0.037 | 0.032-0.043 |
| Optical density of colloid | 0.220 | 0.164-0.327 | 0.237 | 0.170-0.274 | 0.176 | 0.118-0.284 |
| Compactness of colloid | 1.57 | 1.50-1.68 | 1.60 | 1.51-1.69 | 1.55 | 1.52-1.64 |
