# Supplementary material for: Investigation of adrenal and thyroid gland dysfunction in dogs with ultrasonographic diagnosis of gallbladder mucocele formation
Source: PLoS One. 2019 Feb 27;14(2):e0212638. doi: 10.1371/journal.pone.0212638 (PMC6392329; doi:10.1371/journal.pone.0212638)
Supplement: S1 Fig — (DOCX) [file pone.0212638.s006.docx]

**Supporting information**

**

**

**SFig 1. Measurements of serum cortisol concentration before and 1 hour after administration of cosyntropin to control dogs and dogs diagnosed with gallbladder mucocele formation.** Horizontal dashed line labeled “a” denotes post-cosyntropin serum cortisol concentration commonly accepted as suggestive of hyperadrenocorticism in dogs. Horizontal dashed line labeled “b” denotes the upper limit of the reference interval established by post-cosyntropin serum cortisol concentrations measured in control dogs in this study. Open circle data points represent dogs with post-cosyntropin cortisol concentrations above either established reference range.
